# Cross-talk of RNA modification "writers" describes tumor stemness and microenvironment and guides personalized immunotherapy for gastric cancer

**DOI:** 10.18632/aging.204802

**Published:** 2023-06-14

**Authors:** Zhuoqi Li, Xuehong Zhang, Wenjie Weng, Ge Zhang, Qianwen Ren, Yuan Tian

**Affiliations:** 1 Radiotherapy Department, Shandong Second Provincial General Hospital, Shandong University, Jinan, China; 2 Key Laboratory of Carcinogenesis and Translational Research (Ministry of Education/Beijing), Division of Etiology, Peking University Cancer Hospital and Institute, Peking University, Beijing, China; 3 Department of Otolaryngology-Head and Neck Surgery, Shandong Provincial ENT Hospital, Shandong University, Jinan, China

**Keywords:** tumor microenvironment, gastric cancer, RNA modification, WM_Score, immunotherapy

## Abstract

Background: RNA modifications, TME, and cancer stemness play significant roles in tumor development and immunotherapy. The study investigated cross-talk and RNA modification roles in the TME, cancer stemness, and immunotherapy of gastric cancer (GC).

Methods: We applied an unsupervised clustering method to distinguish RNA modification patterns in GC. GSVA and ssGSEA algorithms were applied. The WM_Score model was constructed for evaluating the RNA modification-related subtypes. Also, we conducted an association analysis between the WM_Score and biological and clinical features in GC and explored the WM_Score model’s predictive value in immunotherapy.

Results: We identified four RNA modification patterns with diverse survival and TME features. One pattern consistent with the immune-inflamed tumor phenotype showed a better prognosis. Patients in WM_Score high group were related to adverse clinical outcomes, immune suppression, stromal activation, and enhanced cancer stemness, while WM_Score low group showed opposite results. The WM_Score was correlated with genetic, epigenetic alterations, and post-transcriptional modifications in GC. Low WM_Score was related to enhanced efficacy of anti-PD-1/L1 immunotherapy.

Conclusions: We revealed the cross-talk of four RNA modification types and their functions in GC, providing a scoring system for GC prognosis and personalized immunotherapy predictions.

## INTRODUCTION

Gastric cancer (GC) is considered a common cancer type [1]. Genetic alterations include gene mutations and SCNAs that inactivate tumor suppressors and activate oncogenes related to WNT, EMT, PI3K/AKT/mTOR, RTK/PIK, and RTK/RAS/MAPK pathways [2]. Aberrant promoter DNA methylation and histone modification are major epigenetic alterations in GC [2]. RNA modifications regulate gene expression and are considered a promising therapeutic target for GC [3].

RNA modifications are identified on mRNA, miRNA, and lncRNAs [3]. m6A is the most important RNA alteration in eukaryotic cells. “Writers”, “erasers”, and “readers” of m6A add, remove, or recognize m6A-modified sites and change biological processes. m6A is related to various aspects of RNA metabolism and plays a key role in GC development [4]. m6A regulator expression is linked to the diversity of TME [5]. The important m6A “writer” METTL3 facilitates the Epithelial-mesenchymal transition (EMT) program through METTL3/ZMYM1/E-cadherin signaling and promotes angiogenesis and glycolysis in GC through the enhancement of HDGF mRNA stability [6, 7]. The known m1A modification “writers” include TRMT10C, TRMT6, TRMT61A, and TRMT61B [3, 8]. m1A could regulate the structure and stability of tRNA and rRNA. Recent studies have also revealed m1A in eukaryote mRNAs, promoting translation [9, 10]. The m1A “writers” TRMT6 and TRMT61A increase m1A methylation in a subset of tRNAs and increase PPARδ translation for the activation of Hedgehog signaling and driving of self-renewal of liver CSCs and tumorigenesis [11]. The TRMT6/61A complex is overexpressed in bladder cancer, causing increased m1A modification on tRFs and dysregulation of the tRF targetome [12]. APA generates transcript isoforms in coding regions or 3’UTRs [13]. CPSF, CSTF, CFI, PCF11, CLP1, and NUDT21 form the core pre-mRNA 3’end processing complex. PABPN1 controls RNA transcripts’ poly(A) tail length through binding at proximal poly(A) sites [13]. APA events are related to a range of cancers, including GC. A deep sequencing-based approach revealed APA-mediated 3’UTR shortening of 513 genes across the GC genome. The 3’UTR shortening of the oncogene NET1 enhances the activity of transcription and increases GC cell migration and invasion *in vitro* [14]. APA factor NUDT21 is upregulated in GC and promotes tumor growth and metastasis through the upregulation of SGPP2 [15]. Editing of A-to-I RNA is mediated by ADAR enzymes. It is observed that ADAR can convert adenosines to inosines in double-stranded RNA substrates, which leads to codon changes and the diversification of protein functions [16]. The A→I deamination reaction is catalyzed by ADARs, including ADAR, ADARB1, and ADARB2 [16]. Primary GC displays an RNA mis-editing phenotype with ADAR/ADARB1 dysregulation from the genomic gain and loss of the ADAR and ADARB1 genes. This ADAR/ADARB1 imbalance is linked to poor prognosis in GC patients [17].

Emerging evidence highlights the crosstalk between numerous types of RNA modifications. FTO can bind to pre-mRNAs in intronic regions in the proximity of alternatively spliced (AS) exons and poly(A) sites [18]. m6A “writers” TRMT6/61A catalyse the formation of m1A and m6A in tRNAs [19]. Depleting m6A “writers” KIAA1429 and METTL3 induces 3’UTR lengthening several hundreds of mRNAs with over 50% targets [20]. m6A RNA modifications and A-to-I RNA editing negatively correlate [21]. Previous studies have been confined to single RNA modifications in cancer studies.

Immunotherapy using ICIs has rejuvenated tumor immunology. However, tumor efficacy is variable and limited to a subset of cancer patients [22]. Immune infiltrates in the TME play a central role in tumor development [22]. ImmAPA score pipelines have identified tumor-specific ImmAPAs [23]. Investigation of the integrated cross-talk of multiple RNA modifications during TME cell infiltration and immunotherapy is required to further improve current immunotherapeutic strategies. In this study, expression profiles and clinical information of 1051 GC patients were integrated to identify RNA modification patterns.

## RESULTS

### Genetic and epigenetic variations of RNA modification “writers” in GC

**Figure 1:**
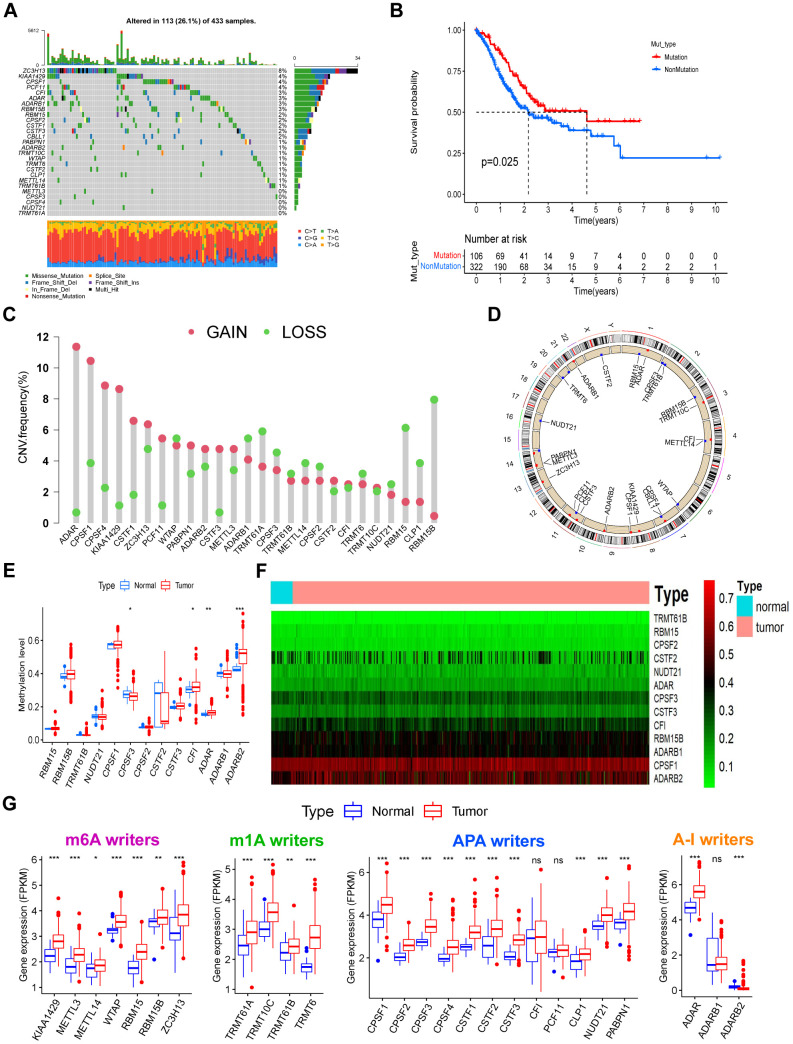
**The landscape of genetic, epigenetic, and transcriptional alterations of RNA modification “writers” in gastric cancer.** (**A**) Mutational frequency of the 26 RNA modification “writers” in 433 GC patients from the TCGA-STAD cohort. Each column represents individual patients. Upper bar plots show TMB, and numbers on the right indicate the mutational frequency in each “writer”. Right bar plots show the proportion of each variant type. Stacked bar plots below show the fraction of conversions in each sample. (**B**) Kaplan–Meier curve showing the overall survival status of patients with mutations (red curve) in one or more of the 26 writers or without mutations (blue curve) in the TCGA-STAD cohort. The grouping status of the STAD patients is indicated at the bottom of the chart. P < 0.05 in the Log-rank test was considered statistically significant. (**C**) CNV variation frequency of the 26 writers in the TCGA-STAD cohort. The height of the column represents the alteration frequency. Red dots represent deletion frequency; blue dots represent amplification frequency. (**D**) Location of the CNV alterations of RNA modification writers on 23 chromosomes from the TCGA cohort. (**E**) The methylation level of some writers between normal and gastric cancer tissues. In the box plot, blue represents normal tissues, and red represents cancer tissue. The upper and lower ends of the boxes represent the interquartile ranges of values. Lines in the boxes represent median values. Blue or red dots show outliers. Asterisks above the boxes represent the p-value (*P < 0.05; **P < 0.01; ***P < 0.001). (**F**) Heatmap of the methylation level of some writers. Methylation levels of these writers increase gradually with the color changes from green to red. (**G**) Expression levels of the 26 writers were composed of four types of RNA modification between normal and gastric cancer tissues. The description of the box plots is as in (**E**).

Twenty-six RNA modification “writers” were investigated. The mutation frequency of the 26 “writers” were first analyzed across pan-cancer in TCGA (Supplementary Figure 2A). Amongst the 433 STAD samples, 113 (26.1%) had mutations in one or more “writers” (Figure 1A). We found ZC3H13 showing the highest mutation rate (8%). Missense mutations and frame-shift deletions were the predominant mutational types. The co-occurrence of mutations was identified in various pairs of genes, including CSTF3 and RBM15, ADARB1 and ZC3H13, and CFI and PCF11 (Supplementary Figure 2B and Supplementary Table 2). Patients with mutations in “writers” showed superior overall survival compared with patients lacking mutations (Figure 1B), suggesting they play a functional role in GC. Using GSVA analysis, the underlying mechanisms were related to the activation of tumor suppressor pathways, including P53 signaling, base excision repair, and mismatch repair pathways (Supplementary Figure 2C and Supplementary Table 3). Copy number variation (CNV) frequency and DNA methylation levels were compared, and mRNA expression levels between paired normal and GC tissues were analyzed. Upregulation of most “writers” was observed in tumor tissue than normal tissue (Figure 1G). Through CNV frequency analysis, CNV gains were more frequent than losses for many “writers”, including ADAR, CPSF1, CPSF4, KIAA1429, CSTF1, and CSTF3 (Figure 1C). CNV sites’ locations for all “writers” on the chromosomes can be found in Figure 1D. Some “writers” showed significant upregulation in tumor tissue without a high frequency of CNV gains or CNV losses. Eight “writers” were selected with obvious inconformity between CNV status and mRNA expression. These were divided into four groups based on their CNV status, including normal tissue, tumors with CNV gains, CNV losses, and non-CNV alterations. We compared the expression levels of the eight “writers” between the four groups (Supplementary Figure 2D–2K). Patients with CNV gains showed significantly higher expression of all “writers,” excluding ADARB2. Besides ADARB2, the expression of all “writers” were similarly upregulated in tumors with no CNV alterations and upregulated or not significantly changed in tumors with CNV losses. The expression of ADARB2 was downregulated in all three tumor groups compared with normal samples regardless of CNV status. Thus, expression levels may not be regulated by CNV alterations. DNA methylation analysis for these “writers” identified that the DNA methylation levels of CPSF3 were downregulated, whilst significant upregulation of CFI, ADAR, and ADARB2 in the tumors was observed (Figure 1E, 1F). Considering the mRNA expression of ADARB2 was markedly down regulated in tumors and unrelated to CNV alterations, we hypothesize that the expression of ADARB2 is regulated by hypermethylation in GC. These analyses demonstrate that 26 RNA modification “writers” expression levels are regulated by gene mutations and DNA methylation, which is highly heterogeneous between normal ones and GC tumors.

### RNA modification patterns by the 26 “writers”

**Figure 2:**
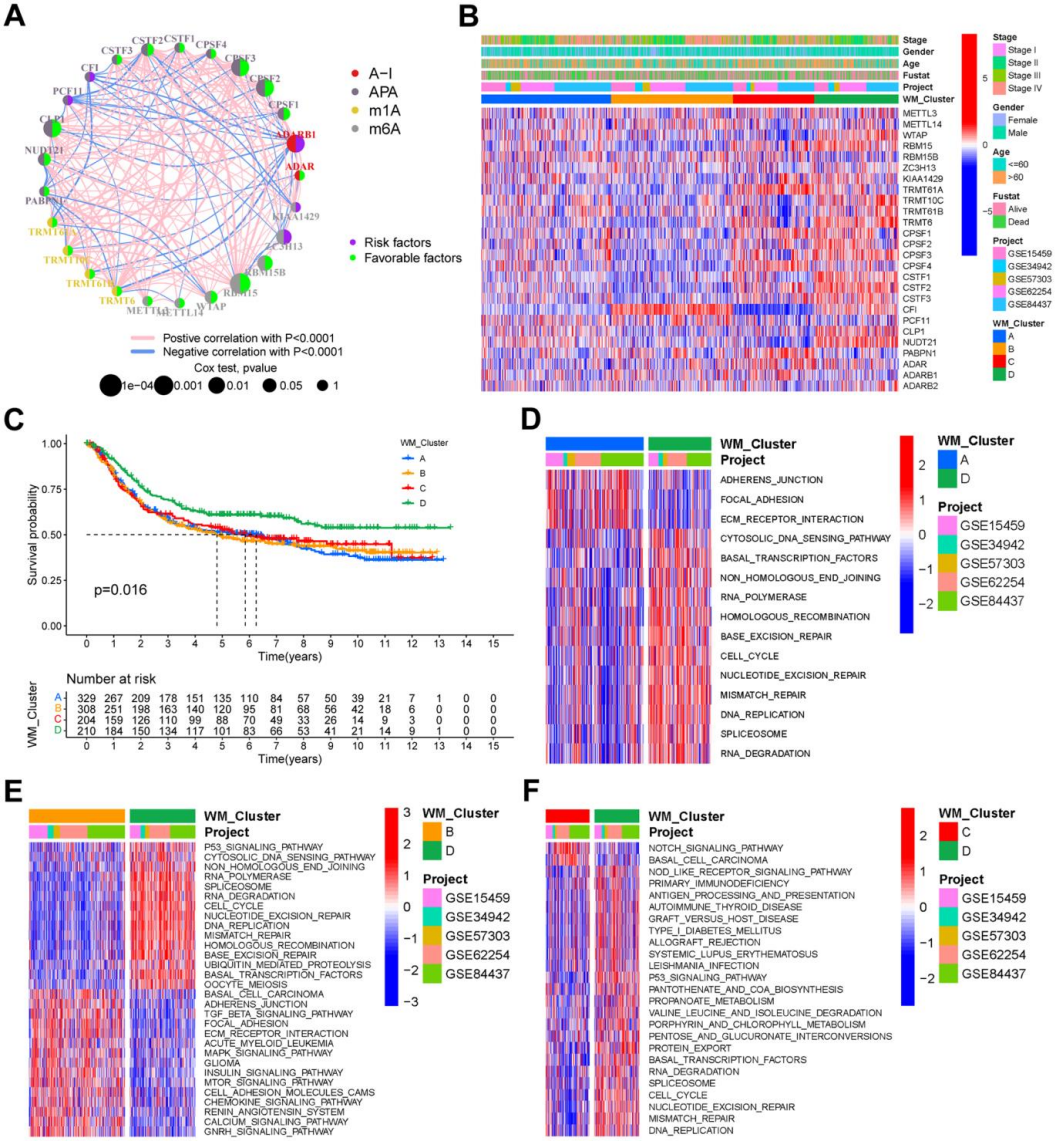
**RNA modification patterns and different biological characteristics between these patterns.** (**A**) Interactions between RNA modification writers in gastric cancer. Circle size represents the effect of each regulator on prognosis, and the range of values calculated by the Log-rank test was p <1e-04, p < 0.001, p < 0.01, p < 0.05, and p < 1, respectively. (**B**) Unsupervised clustering of the 26 writers in the meta-GEO cohort. WM_Cluster, GC cohort names, tumor stage, survival status, age, and gender were used as patient annotations. Red represents a high expression of the writers, and blue represents a low expression. (**C**) Kaplan–Meier curve showing the overall survival status of four types of RNA modification patterns based on 1051 patients of the meta-GEO cohort. WM_Cluster A, blue; WM_Cluster B, orange; WM_Cluster C, red; WM_Cluster D, green. The grouping status of the patients is indicated at the bottom of the chart. P < 0.05 in the Log-rank test was considered statistically significant. (**D**–**F**) GSVA enrichment analysis shows differentially activated biological pathways between the RNA modification patterns. Heatmaps were used to visualize these biological processes. Red represents activated pathways, whilst blue represents inhibited pathways. GC cohort names and WM_Cluster were used as patient annotations. (**D**) WM_Cluster A vs WM_Cluster D; (**E**) WM_Cluster B vs WM_Cluster D; (**F**) WM_Cluster C vs WM_Cluster D.

RNA expression profiles of five GEO gastric cancer cohorts were merged with their clinical data into a single meta-GEO cohort containing 1051 GC patients. In the meta-GEO cohort, we have done Univariate Cox regression analysis to investigate the prognostic value of the 26 RNA modification “writers” for GC (Supplementary Figure 2L). A comprehensive landscape of mutual interactions, regulatory connections, and prognostic significance of the 26 “writers” in GC are presented in a regulatory network (Figure 2A). Within the network, a strong correlation between expressions was observed. TCGA-STAD cohort correlation analysis showed a stronger positive correlation of writer expression (Supplementary Figure 2M) amongst four types of RNA modification “writers”. GO BP enrichment analysis for these “writers” showed involvement in RNA processing and modification (Supplementary Figure 2N). Patients were grouped into distinct RNA modification patterns in accordance with the 26 “writers” in the meta-GEO cohort expressions (Supplementary Figure 3A–3F). Eventually, four distinct types of RNA modification patterns, including 329 patients in pattern A, 308 patients in pattern B, 204 patients in pattern C, and 210 patients in pattern D, were identified. These were named WM_Cluster A-D, respectively (Figure 2B, Supplementary Figure 3G, and Supplementary Table 4). The expression of these “writers” varied greatly between different clusters (Supplementary Figure 3H). Patients in the WM_Cluster D had significantly superior prognoses than patients in the other three patterns (Figure 2C).

### Biological pathways and TME cell infiltration in diverse RNA modification patterns

**Figure 3:**
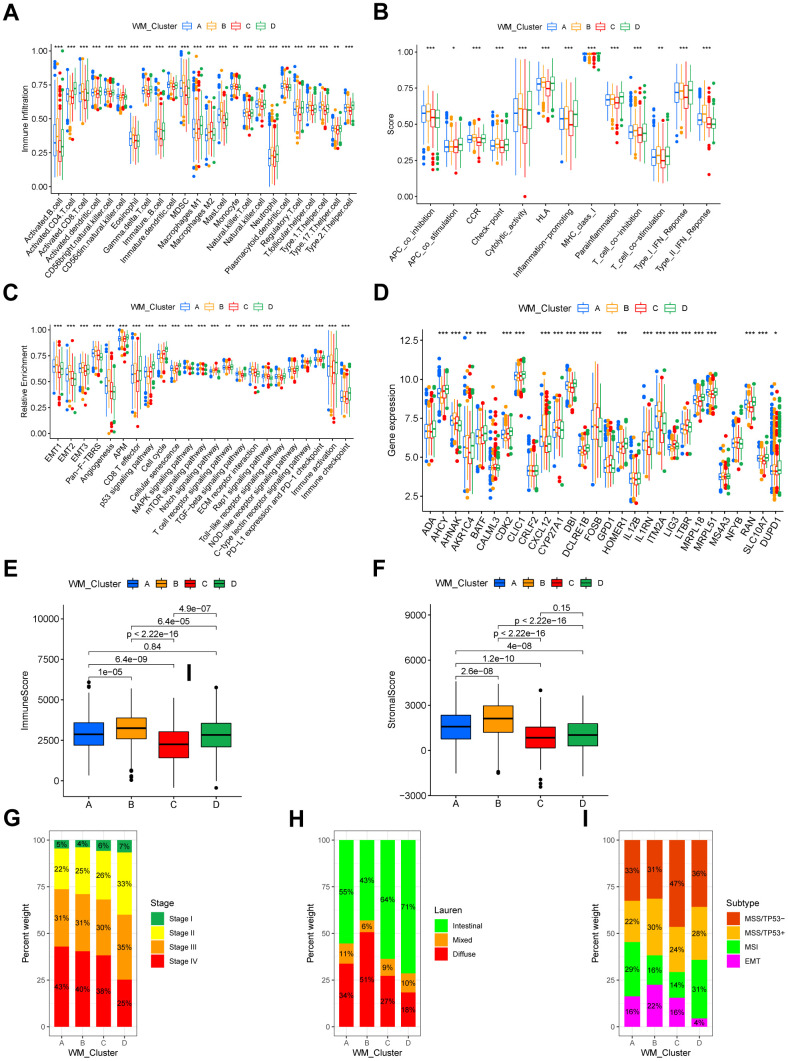
**TME characteristics and clinical features of distinct RNA modification patterns.** (**A**) Relative abundance of 24 TME infiltrating cells in four RNA modification patterns. Names of the infiltrating cells are listed at the bottom of the chart. (**B**) Score of functions in immune regulation of four RNA modification patterns. Immune functions are listed. (**C**) Relative enrichment of stromal-related, immune-related and tumor-related pathways of the four RNA modification patterns. Pathway names are listed below. (**D**) Expression of T cell function enhancers. Gene names of these enhancers are listed. In both the box plots of (**A**–**D**), the color of WM_Cluster A is blue, WM_Cluster B is orange, WM_Cluster C is red, and WM_Cluster D is green. The upper and lower ends of the boxes represent an interquartile range of values. Lines in the boxes represent the median value. Four colors of the dots are outliers. Asterisks above the boxes represent the statistical p-value (*P < 0.05; **P < 0.01; ***P < 0.001). (**E**) Immune score and (**F**) stromal score of four types of RNA modification patterns. P < 0.05 in the Kruskal-Wallis test was regarded as statistically significant. (**G**–**I**) Bar plots showing RNA modification patterns in different clinical stages (**G**), ACRG molecular subtypes (**H**), and Lauren subtypes (**I**).

GSVA analysis was performed for the identification of distinctions in biological pathways between WM_Cluster D and WM_Cluster A-C (Figure 2D–2F and Supplementary Table 6). Pathways of stromal and carcinogenesis activation were inhibited, whilst cell senescence and apoptosis were activated in WM_Cluster D compared with WM_Cluster A-C. ECM receptor interactions were inhibited whilst cell cycle and mismatch repair pathways were activated in WM_Cluster D compared with WM_Cluster A (Figure 2D); TGF-beta signaling, MAPK signaling, and mTOR signaling were suppressed, but P53 signaling, cell cycle, and activation of mismatch repair pathways were observed in WM_Cluster D compared with WM_Cluster B (Figure 2E). Notch signaling and basal cell carcinoma were suppressed whilst P53 signaling, cell cycle, and nucleotide excision repair pathways were activated in the WM_Cluster D compared with the WM_Cluster C (Figure 2F). Surprisingly, samples in the WM_Cluster D were infiltrated with more abundantly immune-active cells, such as CD4 T cells, activated CD8 T cells, activated dendritic cells and macrophage M1 (Figure 3A). Subsequent analysis revealed that an array of immune-related functions was more active in WM_Cluster D (Figure 3B and Supplementary Tables 9, 10). Stromal-related and carcinogenic pathways were also inhibited, and tumor-suppressive and immune-active pathways were activated in the WM_Cluster D (Figure 3C). T cell enhancers were most highly expressed in the WM_Cluster D (Figure 3D). The immune-activation and immune-checkpoint gene expressions were also upregulated, whilst the stromal-activation gene expressions were downregulated in the WM_Cluster D (Supplementary Figure 3J–3L). Patients in the WM_Cluster D showed high immune but low stromal scores (Figure 3E, 3F). The WM_Cluster B was infiltrated by more stromal and eosinophils, MDSCs, mast cells, M2 macrophages, and regulatory T cells (Figure 3A). The WM_Cluster B was strongly associated with stromal activation based on the former GSVA analysis. Stromal-related pathways were significantly activated in the WM_Cluster B (Figure 3C). The WM_Cluster B was also the highest according to stromal score and stromal-related gene expression amongst the four groups (Figure 3F and Supplementary Figure 3L). We speculated that immunosuppressive cell infiltration and stromal activation inhibited antitumor immune responses, so patients in the WM_Cluster B showed no preferential prognosis. WM_Cluster C was the lowest for immune cell infiltration and immune-related functions (Figure 3A–3C). The WM_Cluster C mirrored immune suppression. The WM_Cluster A was intermediate with no significant characteristics of TME infiltration. Correlation analysis between TME infiltration cell type and immune activation showed a high correlation with WTAP, RBM15, NUDT21, CSTF3, and CPSF2 (Supplementary Figure 3I).

### Association between clinical features and RNA modification patterns

We analyzed their association with clinical features. A proportion of tumor stages differed between the four clusters in the meta-GEO cohort, and tumor stages of the patients in the WM_Cluster D were less advanced (Figure 3G). Lauren’s subtypes were also related to the WM_Cluster, and the intestinal subtype of GC counts was predominant in the WM_Cluster D, whilst the diffuse subtype counts were lower. Assessment of the GSE62254 ACRG cohort classified GC into four types: EMT, MSI, MSS with TP53-active, and MSS with TP53-inactive. We found that the proportion of the MSI subtype of tumors was highest whilst the proportion of the EMT subtype was lowest in the WM_Cluster D. These results described how patients in the WM_Cluster D have superior overall prognosis over patients in the other three clusters.

### Identifying RNA modification-associated genes and assessment of their functions

**Figure 4:**
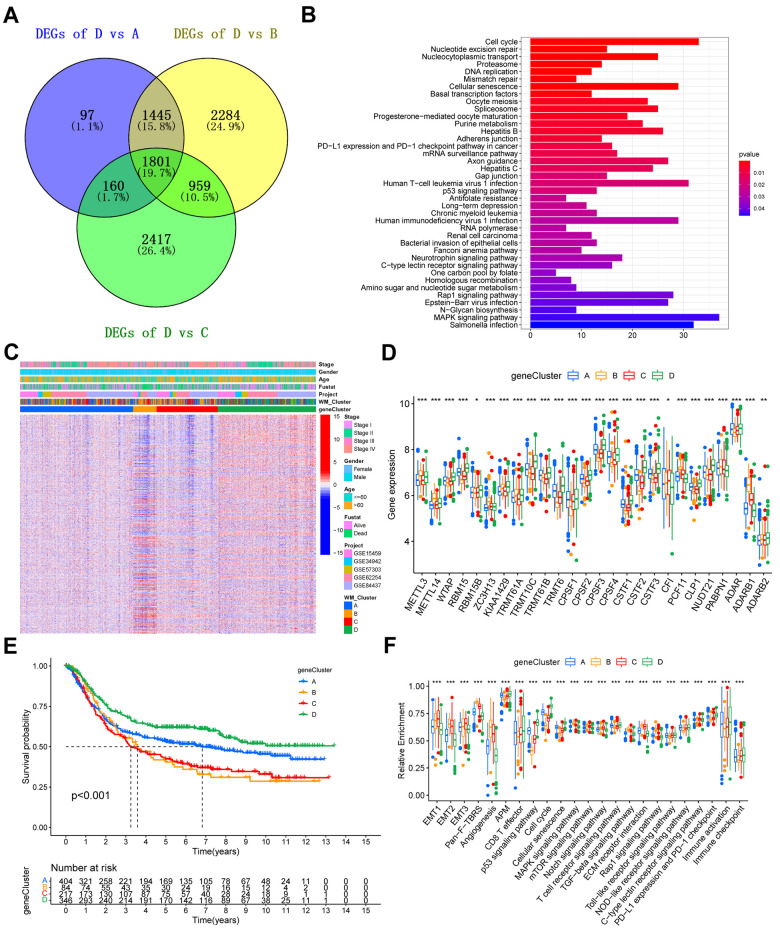
**Construction of gene signatures and functional annotation.** (**A**) Venn diagram showing the 1801 overlapped DEGs between WM_Cluster D and the other three clusters. (**B**) KEGG enrichment analysis of the overlapped 1801 RNA modification patterns. The length of the bars represents the number of genes enriched. (**C**) Unsupervised clustering of overlapping RNA modification-related genes in the meta-GEO cohort to classify patients into different genomic subtypes, defined as gene clusters A-D, respectively. Gene clusters, WM_Clusters, GC cohort names, tumor stage, survival status, age, and gender were used as patient annotations. (**D**) Kaplan–Meier curve showing the overall survival status of the four gene clusters based on the meta-GEO cohort. Gene cluster A, blue; gene cluster B, orange; gene cluster C, red; gene cluster D, green. The grouping status of the patients is indicated at the bottom of the chart. P < 0.05 in the Log-rank test was considered statistically significant. (**E**) Expression levels of the 26 writers in the four gene clusters. (**F**) Relative enrichment of some stromal-related, immune-related, and tumor-related pathways of the four gene clusters. For (**E**) and (**F**), the upper and lower ends of the boxes represent interquartile ranges of values. Lines in the boxes represent the median value. The four colors of the dots are outliers. Asterisks above the boxes represent the statistical p-value (*P < 0.05; **P < 0.01; ***P < 0.001).

We identified 1801 DEGs common between WM_Cluster D and the other three clusters (Figure 4A). The biological functions of these DEGs were assessed. Genes were enriched in pathways related to cell cycle and cell senescence, PD-L1 expression, PD-1 checkpoints, carcinogenesis, and cancer (Figure 4B and Supplementary Table 14). Unsupervised clustering analysis was done based on the expression of the 1801 DEGs were performed to classify patients into different genomic subtypes (Supplementary Figure 4A–4F). We identified four distinct genomic subtypes and termed them gene clusters A-D, respectively (Figure 4C and Supplementary Table 4). Differences in the expression of the 26 “writers” between the four gene clusters were to the four RNA modification patterns (Figure 4D). Patients in gene cluster D had the highest overall survival, consistent with the RNA modification patterns’ prognosis analysis (Figure 4E). Mechanistically, stromal-related and carcinogenic pathways were significantly inactivated, whilst pathways for anti-tumor immune activation, cell senescence, and apoptosis were highly activated in gene cluster D (Figure 4F). T cell function enhancer and immune activation genes were also highly expressed, whilst genes regulating stromal activation were downregulated (Supplementary Figure 4I, 4J). Patients in gene cluster D also benefited from immune-checkpoint block therapy (Figure 4F and Supplementary Figure 4I).

### Quantifying RNA modification patterns

**Figure 5:**
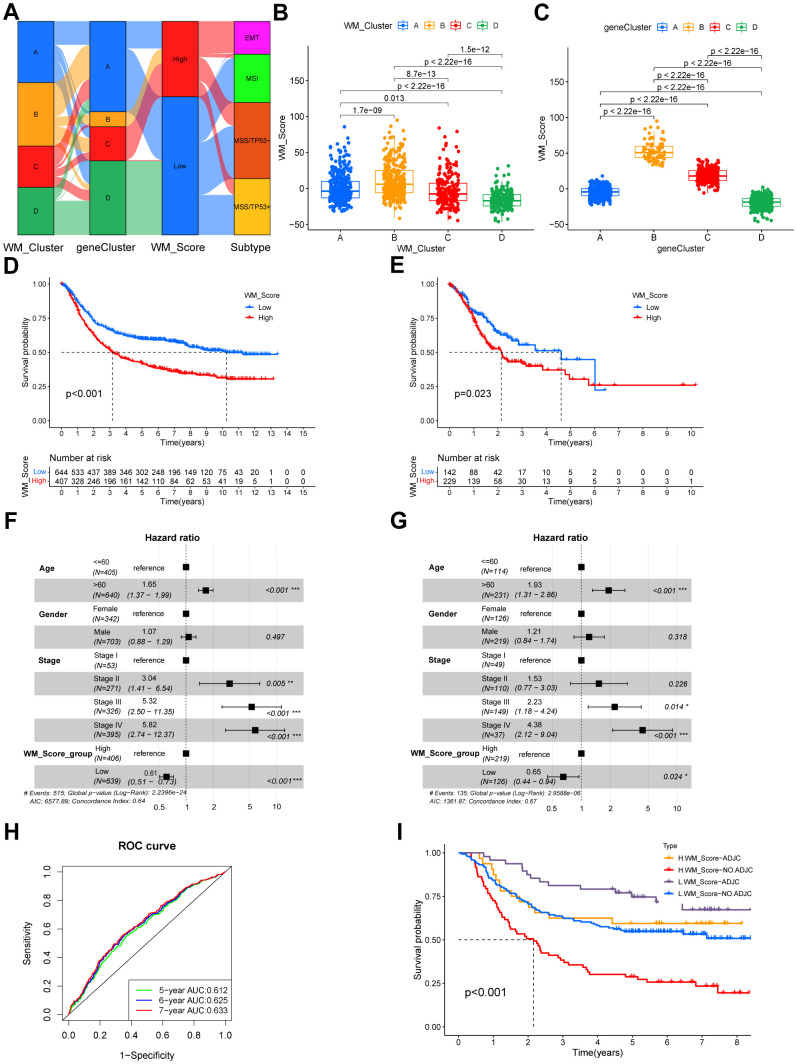
**Quantifying RNA modification patterns according to WM_Score.** (**A**) Sankey plot showing the relationship between WM_Clusters, gene cluster, WM_Score and ACRG molecular subtypes. (**B**, **C**) WM_Scores according to different RNA modification patterns and gene clusters in the meta-GEO cohort. P < 0.05 in the Kruskal-Wallis test was considered statistically significant. (**D**, **E**) Kaplan–Meier curves showing the overall survival status of WM_Score-low (blue) and WM_Score-high (red) groups of patients in the meta-GEO cohort (**D**) and TCGA-STAD cohort (**E**), grouping status of the patients are indicated at the bottom of the chart. P < 0.05 in the Log-rank test was considered statistically significant. (**F**) Kaplan–Meier curves of WM_Score patients with or without adjuvant chemotherapy. H, high; L, low; ADJC, adjuvant chemotherapy. P < 0.001 by Log-rank test. (**G**, **H**) Multivariate Cox regression model of WM_Score, patient age, gender, TNM status, and patient outcomes in the meta-GEO cohort (**G**) and TCGA-STAD cohort (**H**) shown by the forest plots. (**I**) Predictive value of the WM_Score in patients amongst the meta-GEO cohort. The AUC of five, six, and seven-year survival was 0.612, 0.625, and 0.633, respectively.

We termed the scoring system as a WM_Score (Supplementary Table 4). Connections among RNA modification patterns, gene clusters, WM_Score groups, and ACRG subtypes in the GSE62254 cohort are shown through a Sankey diagram (Figure 5A). Consistent with previous data, we classified the majority of patients in WM_Cluster D into gene cluster D and WM_Score low groups. No patients in the WM_Score low group were classified into the EMT subtype (Figure 5A). The WM_Score was found to vary across different RNA modification patterns. WM_Cluster B had the highest WM_Score, whilst WM_Cluster D had the lowest (Figure 5B). Consistently, the WM_Score was highest in gene cluster B but lowest in gene cluster D (Figure 5C).

### Biological and clinical traits related to the WM_Score

In prognostic analysis, low WM_Score group patients had a significantly higher survival status than the high WM_Score group patients in the meta-GEO cohort (Figure 5D). The WM_Score can predict patient prognosis according to age, gender, and tumor stage (Figure 5F). We used AUCs of ROC curves for the WM_Score to predict five, six, and seven-year survival rates of 0.612, 0.625, and 0.633, respectively (Figure 5G), demonstrating the WM_Score as a robust and independent tool for the prediction of patient prognosis. We validated WM_Score’s readability in the TCGA-STAD cohort. Consistent with the results, patients in the TCGA-STAD cohort were grouped into high and low WM_Score groups (Supplementary Table 5). Low WM_Score patients had preferable overall survival status following univariate (Figure 5E) and multivariate (Figure 5G) Cox regression analysis. Furthermore, a low WM_Score and adjuvant chemotherapy showed an improved prognosis compared to patients with a high WM_Score or no adjuvant chemotherapy (Figure 5I).

**Figure 6:**
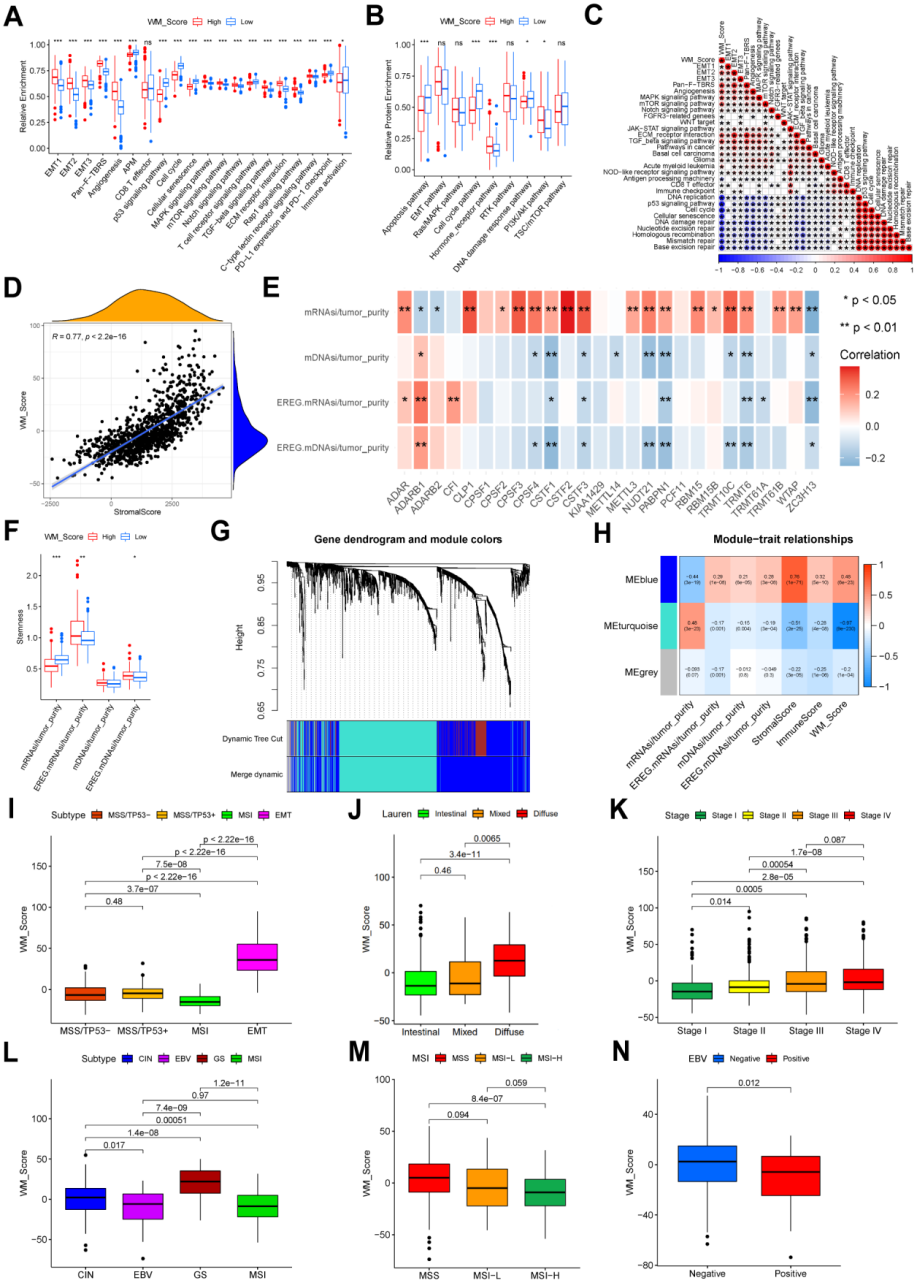
**Relevance between WM_Score, biological characteristics, and clinical features.** (**A**) Relative enrichment of stromal-related, immune-related and tumor-related pathways of four RNA modification patterns. Pathway names are listed below. (**B**) Relative protein enrichment levels of biological pathways. In (**A**, **B**), the color of the WM_Score-low group is blue, WM_Score-high group is red. The upper and lower ends of the boxes represent interquartile ranges of values. Lines in the boxes represent median values. Blue and red dots are outliers. Asterisks above the boxes represent the p-value (*P < 0.05; **P < 0.01; ***P < 0.001). (**C**) Correlation between WM_Score and known biological pathway gene signatures using Spearman analysis. A negative correlation is shown in blue; a positive correlation is in red. (**D**) Spearman correlation analysis between WM_Score and stromal score (R = 0.77, p < 2.2e-16). (**E**) Spearman correlation analysis between each ratio of stemness index to tumor purity and each RNA modification writers. Negative correlation: blue; positive correlation: red. (**F**) Differences in stemness indices between WM_Score high and low groups (*P < 0.05; **P < 0.01; ***P < 0.001). (**G**) Gene dendrogram of the identified co-expressed genes in different modules. Branches of the dendrogram correspond to the different gene modules. Each leaf on the dendrogram represents a gene. Each block marked by a color represents a module that contains a group of highly correlated genes. A total of four dynamic modules and three merged modules were identified. (**H**) Correlation between gene modules and clinical traits. The correlation coefficient and corresponding p-value are annotated in the blocks of the module-trait relationships map. Positive correlation: red; negative correlation: blue. (**I**) Different WM_Scores between ACRG molecular subtypes. (**J**) Different WM_Scores between Lauren subtypes. (**K**) WM_Scores according to the clinical stages of patients in the meta-GEO cohort. (**L**) Different WM_Scores in different TCGA-STAD molecular subtypes. (**M**) Different WM_Scores amongst different MSI statuses of patients in the TCGA-STAD cohort. (**N**) Different WM_Scores amongst patients with or without EBV infection in the TCGA-STAD cohort. In (**I**–**N**) Upper and lower ends of the boxes represent interquartile ranges. Lines in the boxes represent median values. Kruskal-Wallis tests were used to compare significant differences between each group.

In this study, we infiltrated the WM_Score low group with more activated CD4 T cells, CD56dim NK cells, and M1 macrophages, whilst the WM_Score high group was infiltrated with cells related to stromal activation and immune suppression, including eosinophils, MDSCs, mast cells, M2 macrophages and regulatory T cells (Supplementary Figure 5A). T cell functional enhancers, immune-activation-related genes, and immune-checkpoint genes were upregulated in the WM_Score low group (Supplementary Figure 5B–5D), but genes related to stromal activation were more downregulated in the low WM_Score group (Supplementary Figure 5E). Following KEGG pathway enrichment analysis, stromal-related and carcinogenic pathways were suppressed, whilst pathways associated with immune activation and cell senescence were active in the low WM_Score group (Figure 6A). The analysis of protein expression data by RPPAs from the TCGA confirmed these data (Figure 6B). The WM_Score is positively related to stromal-related and carcinogenic pathways but negatively correlated with pathways related to immune activation and cell senescence (Figure 6C). The WM_Score also correlated with the stromal score in GC (Figure 6D). The majority of the 26 “writers” positively correlated with mRNAsi but negatively correlated with mDNAsi, EGER.mRNAsi, and EGER.mDNAsi (Figure 6E). Previous results showed that most “writers” were upregulated in the WM_Cluster D, so the stemness indices also varied with different RNA modification patterns. The WM_Cluster D had the highest mRNAsi but the lowest mDNAsi, EGER.mRNAsi, and EGER.mDNAsi. Differences in stemness were observed between the high and low WM_Score groups, and the dedifferentiation phenotype was more obvious in the WM_Score high group compared to the more obvious differentiation phenotype in the WM_Score low group (Figure 6F). The 1801 DEGs of different RNA modification patterns were acted in the analysis, and four corrected stemness indices, immune score, stromal score, and WM_Score, were applied to define phenotypes. To obtain a scale-free network, we selected the soft threshold power β as 5 being 0.9 (Supplementary Figure 6A). Genes with similar expression patterns were inputted into modules through average link clustering (Figure 6G). Finally, three modules were identified (blue, turquoise, and grey; Figure 6H and Supplementary Table 15). We used Module eigengenes (MEs). Genes in the blue module positively correlated with all features, particularly stromal score, and WM_Score, except for mRNAsi. Genes in the turquoise module positively correlated with mRNAsi but negatively correlated with other features, especially stromal score and WM_Score (Figure 6H). According to KEGG enrichment analysis, genes in blue and turquoise modules were enriched in different pathways. Genes in turquoise modules were related to cell senescence and apoptosis, immune activation, immune-checkpoint expression, and DNA repair (Supplementary Figure 6B). Genes in blue modules were enriched in Rap1 signaling, ECM−receptor interactions, and Hedgehog signaling (Supplementary Figure 6C). This confirmed that tumor stemness, stromal activation, and immune activation vary according to RNA modification patterns, which can be quantified according to the WM_Score. We also compared the WM_Score in different clinical subtypes of gastric cancer. In the GSE62254 cohort, the EMT subtype had the worst prognosis and highest WM_Score. The MSI subtype showed the highest prognosis and lowest WM_Score (Figure 6I). The Lauren subtype in the meta-GEO cohort, the diffuse subtype of GC, had the highest WM_Score. The intestinal subtype with the best prognosis had the lowest WM_Score (Figure 6J). The WM_Score increased according to the tumor stage advancing (Figure 6K). Similar results were observed in the TCGA-STAD cohort. The WM_Score was highest in subtypes with the worst prognosis but lowest in subtypes with the best prognosis. The WM_Score was highest in the GS subtype but lowest in the MSI subtype (Figure 6L). The WM_Score decreased with microsatellite instability (MSI), and the high MSI subtype of GC had the lowest WM_Score (Figure 6M). The WM_Score also decreased with EBV infection (Figure 6N).

### WM_Score associated with genetic and epigenetic alterations

**Figure 7:**
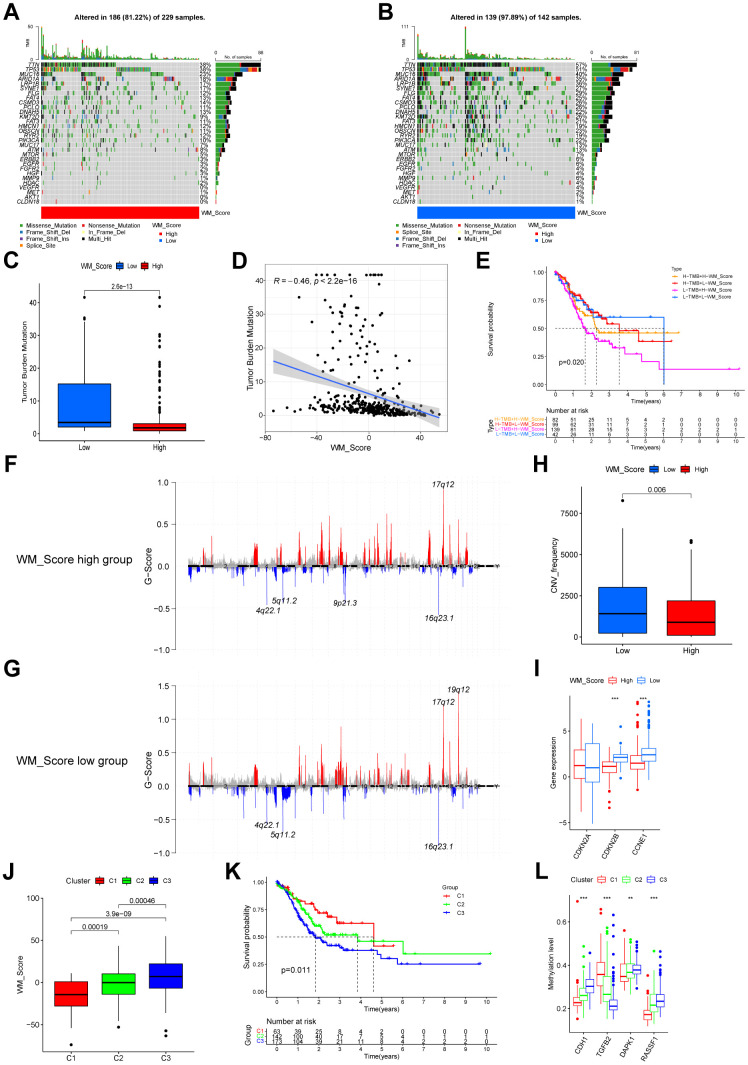
**Relationships between WM_Score and genetic and epigenetic alterations.** (**A**, **B**) Waterfall plot of tumor somatic mutations established by those with high WM_Score (**A**) and low WM_Score (**B**). Each column represents individual patients. Upper bar plots show TMB. The numbers on the right indicate mutation frequency in each gene. The right bar plot shows the proportion of each variant type. (**C**) Difference of TMB between WM_Score high and low groups (Wilcoxon test). (**D**) Correlation between WM_Score and TMB (R = -0.46, p < 2.2e-16, Pearson’s Correlation test). (**E**) Kaplan–Meier curves showing the overall survival status of WM_Score high and low patients with low or high TMB. H, high; L, low. P = 0.02 by Log-rank test. (**F**, **G**) Distribution of copy number amplification and deletion sites in the WM_Score high group (**F**) and WM_Score low group (**G**). The Abscissa axis represents the location of CNV on the chromosome. The ordinate axis represents the G-score. Amplifications are marked with red, and deletions with blue. (**H**) CNV frequency in WM_Score high and low groups (p = 0.006, Wilcoxon test). (**I**) Box plot showing the expression of genes with significant differences in CNVs between the two groups. (**J**) WM_Scores according to DNA methylation subtypes (Kruskal-Wallis test). (**K**) Kaplan–Meier curve showing the overall survival status of three methylation subtypes of patients in the TCGA-STAD cohort (p = 0.011, Log-rank test). (**L**) Box plot showing the methylation level of GC survival-related genes (*P < 0.05; **P < 0.01; ***P < 0.001).

We compared the most frequently mutated genes and known effectors of targeted therapy and found that the mutation rates of all these genes were significantly higher (Figure 7A, 7B). According to TMB quantification analysis, a high WM_Score was significantly associated with a lower TMB (Figure 7C, 7D). Patients with low WM_Scores may therefore benefit more from targeted therapies for GC. Patients with low TMB and high WM_Scores had the worst prognosis than the other three groups (Figure 7E). The distribution of CNVs is shown in Figure 7F, 7G. The general CNV frequency was higher in the low WM_Score group (Figure 7H). The frequency of 9p21.3 deletions was higher in the high WM_Score group, whilst 19q12 amplifications occurred more frequently in the low WM_Score group (Figure 7F, 7G). Two important tumor suppression genes, CDKN2A, and CDKN2B, are transcribed from 9p21.3, the deletion of which contributes to tumorigenesis and tumor development, including GC. Given these data, we considered that TME immune suppression and inactivation of tumor suppressor genes caused by the higher frequency of 9p21.3 deletions might explain the poor prognosis of the low WM_Score group. However, the important oncogene CCNE1 is transcribed from 19q12, so its amplification frequency may be higher in the low WM_Score group. Factors affecting the biological and clinical outcomes were also variable. Expression level analyses for these genes identified CDKN2B and CCNE1 as more significantly highly expressed in the low WM_Score group, consistent with 9p21.3 locus deletion and 19q12 locus amplification (Figure 7I). Unsupervised clustering could divide patients in the TCGA-STAD cohort based on the methylation levels of prognosis-related gene sites (Supplementary Figure 7A–7F). Amongst the three DNA methylation clusters, the WM_Score was unfavorably related to patient prognosis (Figure 7J, 7K). DNA methylation levels of CDH1, DAPK1, and RASSF1 were positively correlated with WM_Score and adversely associated with patient survival, whilst DNA methylation levels of stromal-related TGFB2 negatively correlated with WM_Score but positively associated with patient survival (Figure 7L).

### WM_Score-related transcriptional and post-transcriptional regulation

**Figure 8:**
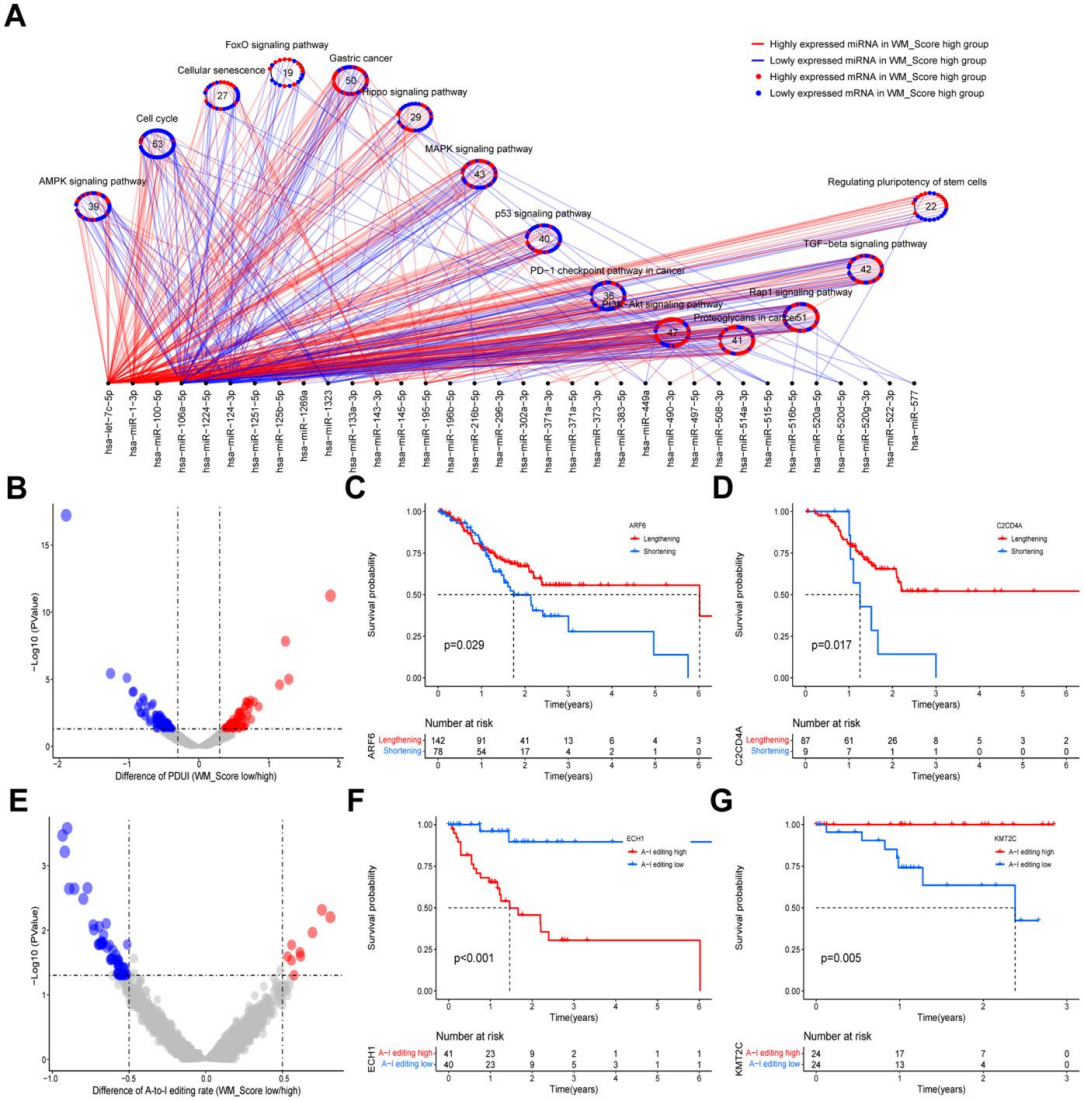
**Relationships between WM_Score and transcriptional and post-transcriptional regulation.** (**A**) Differentially expressed miRNAs and their associated pathways between WM_Score high and low groups. Red lines and dots represent highly expressed genes in the WM_Score high group; blue lines and dots represent highly expressed genes in the WM_Score low group. Circles represent targeted mRNA-enriched pathways. (**B**) Differences in the percentage of Distal poly(A) site Usage Index (PDUI) of the genes between WM_Score high and low groups. Red, PDUI lengthened genes in WM_Score high group; blue, PDUI shortened genes in WM_Score high group; grey, genes with no significant difference in PDUI. (**C**, **D**) Kaplan–Meier curves showing the overall survival status of PDUI lengthening (red) and PDUI shortening (blue) of ARF6 (**C**) and C2CD4A (**D**). (**E**) Differences of A-I editing frequency of genes between WM_Score high and low groups. Red, high A-I editing genes in the WM_Score high group; blue, low A-I editing genes in WM_Score high group; grey, genes with no significant difference in A-I editing. (**F**, **G**) Kaplan–Meier curves showing the overall survival status of A-I editing high (red) and A-I editing low (blue) of gene ECH1 (**F**) and SAMHD1 (**G**).

To investigate the effects of the WM_Score on transcriptional and post-transcriptional regulation, we studied the miRNA regulation to bridge the investigation of these two aspects in the TCGA-STAD cohort. We first screened 112 differentially expressed miRNAs (DEmiRNAs) and 8480 mRNAs (differentially expressed) between high and low WM_Score groups. We then predicted the target genes of the differentially expressed miRNAs using the miRTarBase database. KEGG enrichment analysis for the target genes was then performed amongst DEmRNAs. The enrichment of target genes was observed in cell cycle, cell senescence, PD-1 checkpoint, and cancer-related pathways (Figure 8A and Supplementary Table 16). Most genes related to tumor suppressor pathways, such as cell cycle status, cell senescence, P53 signaling, AMPK signaling, and Hippo signaling, were expressed to low levels in the high WM_Score group. Genes associated with the PD-1 checkpoint pathway were also downregulated in the high WM_Score group. These genes were mainly regulated by highly expressed miRNAs in the high WM_Score group (Figure 8A). Genes upregulated in the WM_Score high group were related to carcinogenic pathways, including gastric cancer, MAPK signaling, PI3K-Akt, proteoglycans in cancer, Rap1 signaling, and TGF-beta signaling. These genes are targeted by low-expressing miRNAs in the high WM_Score group (Figure 8A).

In this study, we identified genes with significantly diverse 3’UTR lengths caused by APA in the high and low WM_Score groups and analyzed their association with patient prognosis (Figure 8B and Supplementary Table 17). We found that ARF6 and C2CD4A showed significant 3’UTR shortening in the WM_Score high group (Supplementary Table 17), which was associated with poor prognosis (Figure 8C, 8D). In our analyses, we speculated that the shortening of mRNA 3’UTRs of these genes in the high WM_Score group could decrease miRNA targeting efficacy and upregulate the expression of these oncogenes, contributing to tumorigenesis and GC development. We also screened out genes with significant differential A-to-I editing rates and analyzed their association with patient prognosis (Figure 8E and Supplementary Table 18). The A-to-I editing rate of ECH1 was higher, whilst that of KMT2C was lower in the high WM_Score group (Supplementary Table 18), which was significantly related to poorer survival (Figure 8F, 8G).

### Predictive value of the WM_Score model in immunotherapy

**Figure 9:**
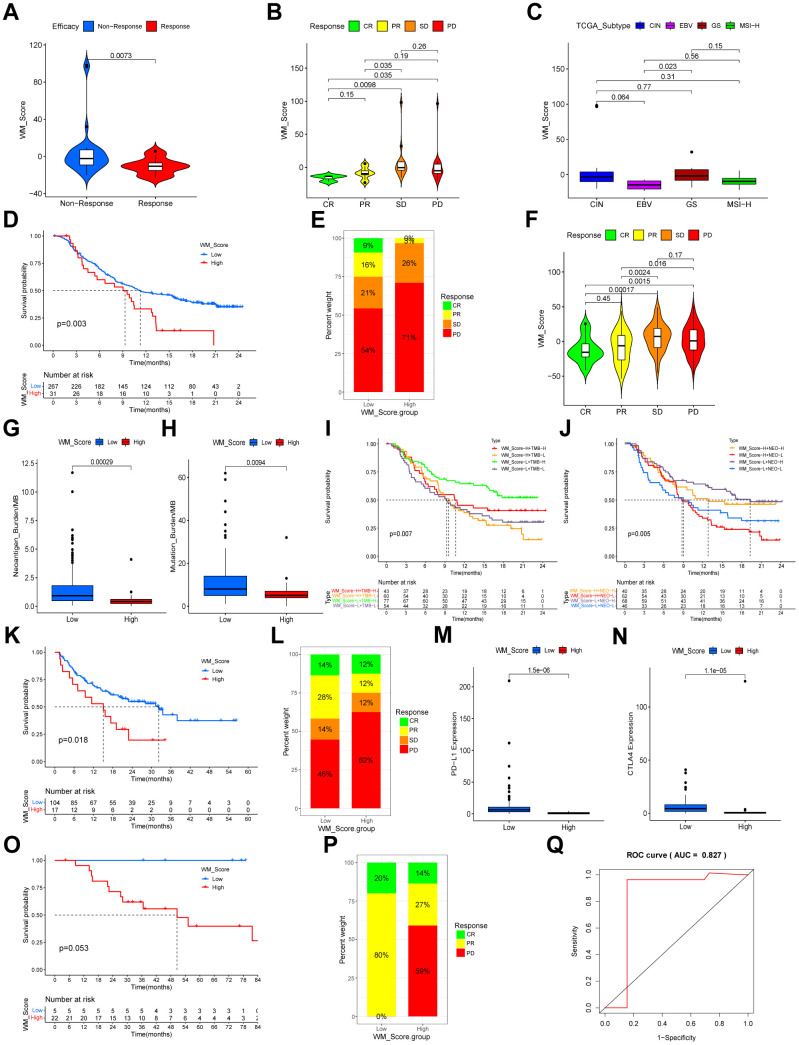
**Relationship between WM_Score and efficacy of immunotherapy.** (**A**, **B**) Violin plots showing the WM_Score in patients with different responses to PD-L1 blockade in the Kim et al. immunotherapy cohort. Kruskal-Wallis tests were used to evaluate the significance. (**C**) Box plot showing the WM_Score in patients of different TCGA subtypes (Kruskal-Wallis test). (**D**) Kaplan–Meier curve showing the overall survival status of patients receiving PD-L1 blockade immunotherapy in the IMvigor210 cohort (p = 0.003, Log-rank test). (**E**) The proportion of patients responding to PD-L1 blockade immunotherapy in low and high WM_Score groups in the IMvigor210 cohort. SD, stable disease; PD, progressive disease; CR, complete response; PR, partial response. (**F**) Violin plot showing the WM_Score in patients with different responses to PD-L1 blockade immunotherapy in the IMvigor210 cohort. (**G**, **H**) Box plots showing the neoantigen burden (**G**) and mutational burden (**H**) of high and low WM_Score groups in the IMvigor210 cohort. Wilcoxon tests were used to evaluate the significance. (**I**) Kaplan–Meier curve showing the overall survival of high or low WM_Score patients with high or low TMB (p = 0.007, Log-rank test). (**J**) Kaplan–Meier curves showing the overall survival of high or low WM_Score patients with high or low neoantigen burden in the IMvigor210 cohort (p = 0.005, Log-rank test). (**K**) Kaplan–Meier curve showing the overall survival status of patients receiving PD1 blockade immunotherapy in high and low WM_Score groups in the Liu et al. immunotherapy cohort (p = 0.018, Log-rank test). (**L**) The proportion of patients responding to PD1 blockade immunotherapy in low and high WM_Score groups. SD, stable disease; PD, progressive disease; CR, complete response; PR, partial response. (**M**, **N**) Box plots showing the expression of PD-L1 (**M**) and CTLA4 (**N**) of high and low WM_Score groups. Wilcoxon tests were used to evaluate significance. (**O**) Kaplan–Meier curve showing the overall survival status of patients receiving anti-PD1 immunotherapy in high and low WM_Score groups in the GSE78220 cohort (p = 0.053, Log-rank test). (**P**) The proportion of patients responding to anti-PD1 immunotherapy in low and high WM_Score groups in the GSE78220 cohort. PD, progressive disease; CR, complete response; PR, partial response. (**Q**) Predictive value of the WM_Score in patients receiving anti-PD-1 immunotherapy in the GSE78220 cohort (AUC = 0.827).

In the Kim et al. cohort, patients in the response group had a lower WM_Score (Figure 9A, 9B). Consistent with his study, EBV-positive and MSI-H subtypes of GC were lower according to WM_Score in this study (Figure 9C). This GC cohort was the only sample group receiving ICB therapy from which gene expression profiles and the responses of immunotherapy could be obtained. We could not identify the survival information of the patients and were unable to make predictions for patient prognosis. We adopted three cohorts of ICB immunotherapy for other cancer types with accessible survival information. Patients in the WM_Score low group exhibited better overall prognosis in both the IMvigor210 cohort (Figure 9D), Liu et al. cohort (Figure 9K), and GSE78220 cohort (Figure 9O). Patients with significant therapeutic advantages and clinical responses to anti-PD-1/L1 immunotherapy also tended to have lower WM_Score in both cohorts (Figure 9E, 9F, 9P). To explore the mechanisms of the superior clinical outcomes in immunotherapy for the low WM_Score group, we analyzed TME cell infiltration, immune and stromal-related gene expression, and pathway enrichment in the two groups in the IMvigor210 cohort. Consistent with our previous findings, the low WM_Score group was infiltrated with a higher number of immune-active cells, such as activated CD4 and CD8 cells and M1 macrophages, but fewer stromal-activation cells such as eosinophils, MDSCs, mast cells and regulatory T cells (Supplementary Figure 8A). An array of T cell enhancer genes and immune activation and immune checkpoint genes were up regulated in the low WM_Score group (Supplementary Figure 8B, 8D, 8E), while stromal-activation-related genes were downregulated (Supplementary Figure 8F). Stromal-related and carcinogenic pathways were inhibited, whilst pathways related to immune activation and cell senescence were activated in the low WM_Score group (Supplementary Figure 8C). Moreover, the inflamed immune phenotype of the tumors had the lowest WM_Score compared with immune excluded and immune desert phenotypes (Supplementary Figure 8G). The expression of PD-L1 on tumor (TC, detected by SP142) and immune cells (IC, detected by SP142) correlated with the WM_Score. TC1 and TC2+ had lower WM_Scores than TC0, whilst IC2+ had significantly lower WM_Scores than IC0 and IC1 (Supplementary Figure 8H, 8I). Patients in the low WM_Score group showed higher expression levels of immune-checkpoint genes PD-L1 and CTLA4 (Figure 9M, 9N). This was consistent with the efficacy of immunotherapy, which was positively associated with PD-L1 expression. A low WM_Score was related to higher tumor neoantigen burden and tumor mutational burden (Figure 9G, 9H). Patients with low WM_Scores and high TMB or high neoantigen levels had a higher survival advantage over other groups (Figure 9I, 9J). The WM_Score is a robust model for clinical responses in immunotherapy (Figure 9Q). The mechanisms underlying its predictive ability were related to TME cell infiltration and PD-L1/PD-1-related pathway enrichment.

### Validating key genes of RNA modification and TME at the protein level

**Figure 10:**
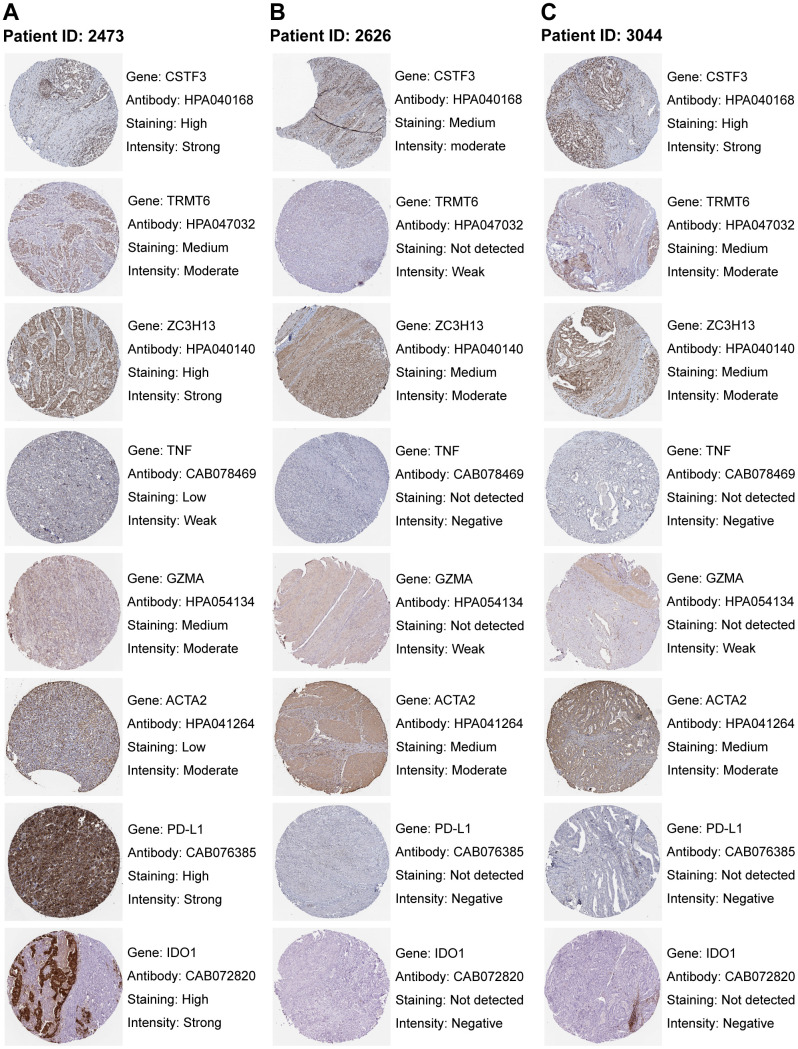
**Staining patterns of RNA modification writers, immune-related genes, stromal-related genes, and immune checkpoints.** (**A**–**C**) Immunohistochemical images of patient ID 2473 (**A**), 2626 (**B**), and 3044 (**C**). Images were downloaded from The Human Protein Atlas (HPA). Genes, antibodies, and staining degree and intensity are listed on the right of each image.

We analyzed GC immunohistochemistry images of three RNA modification “writers”, two immune activation-related genes, one stromal activation-related gene, and two immune checkpoints of three patients in the HPA database. Each gene among the three patients was stained using identical antibodies. CSTF3, TRMT6, and ZC3H13 were higher expressed in WM_Cluster D (Supplementary Figure 3H), which was associated with immune activation, stromal inhibition, and activation of PD-L1/PD-1 checkpoints (Figure 3C). In the images, CSTF3, TRMT6, and ZC3H13 expression were higher in the GC tissues of patient 2473 compared to patients 2626 and 3044 (Figure 10A–10C). Therefore, the RNA modification patterns of patient 2473 were more closely to WM_Cluster D. TNF, and GZMA were more highly expressed in patient 2473, but ACTA2 was expressed at lower levels. Surprisingly, the expression of PD-L1 and IDO1 was only detected in patient 2473 (Figure 10A–10C).

### Exploring WM_Score performance across pan-cancer types

**Figure 11:**
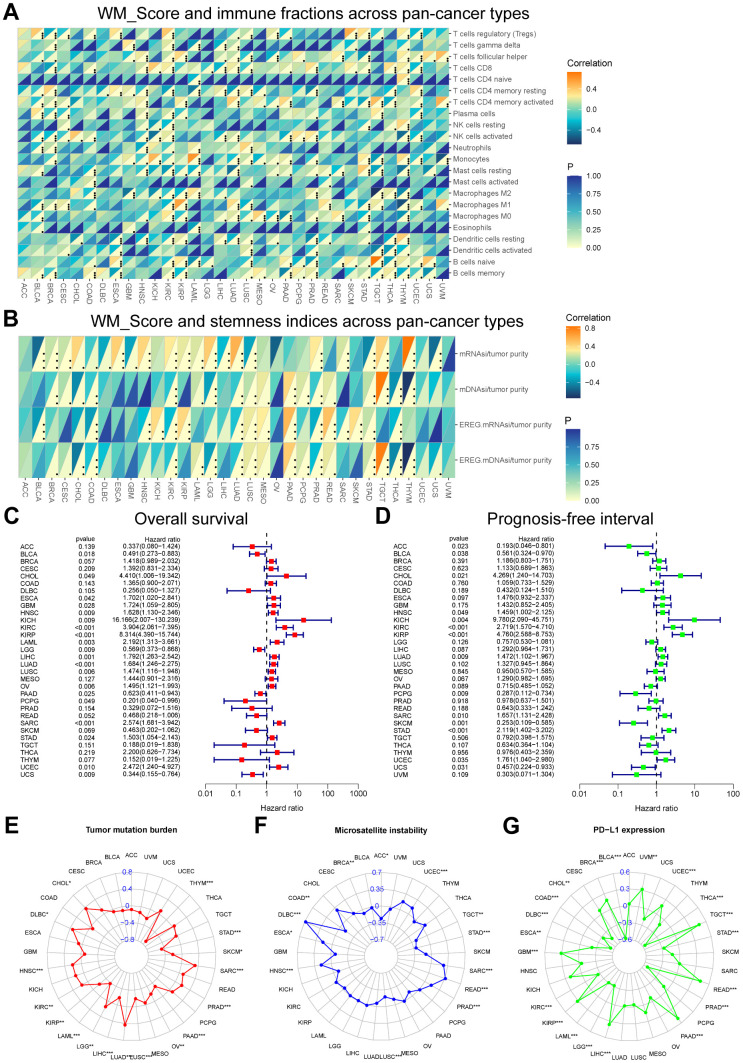
**Performance of the WM_Score across pan-cancer types.** (**A**, **B**) Correlation between WM_Score and immune cell fractions (**A**) and stemness indices (**B**) for each cancer type. Upper regions of grids show p-values. The bottom regions show the correlation coefficient. (**C**, **D**) Overall survival (**C**) and prognosis-free interval (**D**) analyses for the WM_Score in TCGA pan-cancer types using a univariate Cox regression model. A hazard ratio < 1 represents protective factors for survival, while hazard ratio > 1 represents risk factors for survival. (**E**–**G**) Radar plots of the correlation between WM_Score and tumor mutation burden (**E**), microsatellite instability (**F**), and PD-L1 expression level (**G**). Dots in the radar plots represent the R-value of correlation: R > 0, positive correlation, and R < 0, negative correlation. Asterisks or the black dots represent the statistical p-value (Pearson’s Correlation test, *P < 0.05; **P < 0.01; ***P < 0.001).

The WM_Score correlated with the infiltrating fraction of 22 immune cell types in nearly all cancer types except for ACC, and the correlation trend for each infiltrating immune cell type varied amongst diverse cancer types (Figure 11A). The correlation trends between the WM_Score and stemness indices were also different across pan-cancer types but generally positively correlated with cancer stemness (Figure 11B). The WM_Score could also predict the overall survival and prognosis-free interval for most cancer types and represented a risk factor for ESCA, LAML, LIHC, LUSC, and UCEC (Figure 11B, 11C). The WM_Score in these cancer types negatively correlated with immune activation-related cell infiltration and positively correlated with stromal activation-related cell infiltration, such as Tregs, follicular helper T cells, and M2 macrophages (Figure 11A) and stemness indices (Figure 11B). TMB, MSI, and PD-L1 expression are biomarkers to evaluate the efficacy of ICB immunotherapy. Correlations between the WM_Score and TMB, MSI, and PD-L1 expression in pan-cancer types are shown using radar charts (Figure 11E–11G).

## DISCUSSION

RNA modifications act in TME immune cell infiltration and the response to immunotherapy. These RNA modifications occur due to the activity of “writers,” which play a key role in tumor immunology and therapy. Previous reports have focussed on the activity of a single “writer” or one RNA modification type. In this study, we 4 major types of RNA modification “writers” and comprehensively analyzed their roles in TME cell infiltration, pathway enrichment, and clinical outcomes of patients with or without ICB immunotherapy in GC.

We identified frequent alterations in genetic, epigenetic, and RNA expression levels of these “writers” in GC. We further identified 4 RNA modification patterns according to the expression levels of the 26 “writers”. We found WM_Cluster D had superior overall survival over the other three RNA modification patterns. The WM_Cluster D was associated with higher immune-activation-related adaptive immune cell infiltration, characterized as the immune-inflamed phenotype of cancer. The WM_Cluster D was more active in CD8 T effectors, T cell receptors, APM, PD-L1 expression, and PD-1 checkpoint pathways. The WM_Cluster B was related to higher stromal and innate immune cell infiltration, characterized as an immune-excluded phenotype of cancer. EMT1/2/3, Pan-F-TBRS, angiogenesis pathways, and stromal-activation-related gene expression were more activated in WM_Cluster B, which validated the TME infiltration analysis, and explained why the WM_Cluster B conferred no survival advantage. WM_Cluster C was associated with low infiltration of nearly all immune cells, characterized as an immune-desert phenotype. No survival advantage was observed due to weak anti-tumor immunology. WM_Cluster A was intermediate with no significant survival characteristics. The expression levels of the 26 RNA modification “writers” were significantly distinct in the WM_Cluster D, and most of the “writers” were more highly expressed in the WM_Cluster D compared with the other three clusters. Some of the “writers” have been shown to facilitate T cell functions. For example, WTAP-mediated m6A modifications could prevent T cells from T cell antigen-receptor (TCR) signaling-induced cell death and promote the activation of T cells by destabilizing ORAI1 and RIPK1 mRNAs [24]. m1A “writer” TRMT61A and TRMT6 can strengthen MYC protein synthesis, which guides naive T cells from a quiescent state into a proliferative state, promoting rapid T cell expansion [25]. However, whether these “writers” were upregulated in the WM_Cluster D and could facilitate the anti-tumor immune functions of T cells and the mechanisms of TME immune activation by the upregulation of these “writers” in GC are rarely reported and require further exploration.

Further analysis showed that the DEGs in WM_Cluster D were also enriched in many cancer-related pathways and PD-L1 expression. Four gene clusters were identified based on the expression of DEGs. This demonstrated how RNA modifications were of great importance in shaping the distinct TME landscape and prognosis of GC. Given that RNA modifications are heterogeneous in individual patients, we constructed a WM_Score model to analyze the relationship between RNA modification patterns, TME landscape, and patient prognosis. High WM_Scores were associated with stromal activation, carcinogenic pathway activation, enhanced cancer stemness, and poor clinical outcomes, whilst low WM_Scores were associated with immune activation, tumor suppression, and improved clinical outcomes. These results were not observed in patients without ICB immunotherapy in the meta-GEO and TCGA-STAD cohort but were validated in four immunotherapeutic cohorts. TGFβ-activated stroma in TME inhibits T-cell responses against tumor cells and tumor susceptibility to anti-PD-1-PD-L1 therapy [26]. Many carcinogenic pathways, including TGF-β, promote the self-renewal and growth of cancer stem cells [27]. From these results. A close relationship between WM_Score, TME characteristics, cancer stemness, ICB immunotherapy, WM_Score, and prognosis of GC patients with and without immunotherapy was observed. Except for immunotherapy, we found that a low WM_Score and adjuvant chemotherapy had a superimposed effect in improving patient prognosis. Some RNA modification “writers” can regulate the sensitivity to chemotherapeutic drugs for certain types of cancer by mediating RNA modifications of some genes. The METTL3/YTHDF2 axis can lead to cisplatin resistance of ovarian cancer by regulating the mRNA stability of IFFO1 in an m6A-dependent manner and inhibiting IFFO1 expression [28]. METTL14 upregulates pri-miR-19a m6A and increases NSLCC resistance to cisplatin through the miR-19a-5p/RBM24/AXIN1 axis [29]. Similar mechanisms mediated by RNA modifications that impact tumor sensitivity to chemotherapy have also been observed in breast cancer [30], bladder cancer [31], and colorectal cancer [32].

The WM_Score is related to many genetic and epigenetic alterations, which are associated with TME features and immunotherapy efficacy. WM_Score negatively relates to TMB, and mutation frequencies of MUC16, KMT2D, and PIK3CA are higher in the low WM_Score group. These gene mutations are associated with high anti-tumor immune cell infiltration, improved overall survival, and enhanced efficacy of PD-L1/PD-1 blockers in GC [33–35]. In the CNV analysis, the frequency of 9p21.3 deletions was higher in the high WM_Score group, which is reported to be related to immune suppression in TME, a poor response to ICB immunotherapy, and poorer prognosis [36, 37]. DAPK1 can enhance anti-tumor immunology, and DNA is hypermethylated in high WM_Score patients, whilst stromal-activation-related genes such as TGFB2 are hypomethylated.

The activation of MAPK, PI3K-Akt, and TGF-beta pathways was done in the high WM_Score high group because of low miRNA expression that target these signaling components. Assessment of the WM_Score model across pan-cancer types identified different predictive values amongst cancer types. Thus, the application of the WM_Score model is cancer-specific and requires further exploration into individual cancer types.

## MATERIALS AND METHODS

### Collection and pre-processing of attainable expression datasets

The workflow is shown in Supplementary Figure 1. Public gene expression results, and relative clinical information of the patients were downloaded from the Gene-Expression Omnibus and Cancer Genome Atlas databases. Patients lacking survival information were removed for further analysis. In total, 5 gastric cancer cohorts in GEO (GSE15459, GSE34942, GSE57303, GSE62254, and GSE84437) and TCGA-STAD, including 1422 patients, were included. Basic information on each dataset is shown in Supplementary Table 1. The “ComBat” algorithm of the sva package [38] was used for the correction of batch effects from non-biological technical bias by R software (version 4.1.3) and R Bioconductor packages.

### Acquisition of the DNA methylation data

DNA methylation data were got from the Cancer Genome Atlas database. The “Methylation Beta Value” was selected. Data from Illumina human methylation 27 and 450 platforms were included.

### Unsupervised clustering of 26 RNA modification writers

An unsupervised clustering algorithm was applied for the cluster analysis of 1051 gastric cancer patients. These RNA “writers” consisted of seven m6A modification enzymes (METTL3, METTL14, WTAP, RBM15, RBM15B, ZC3H13 and KIAA1429), four m1A modification enzymes (TRMT61A, TRMT61B, TRMT10C and TRMT6), twelve APA modification enzymes (CPSF1/2/3/4, CSTF1/2/3, PCF11, CFI, CLP1, NUDT21 and PABPN1) and 3 A-I modification enzymes (ADAR, ADARB1 and ADARB2). The consensus clustering algorithm was used to determine the number of clusters. Stability was assessed using the ConsensusClusterPlus package.

### GSVA and functional annotation

The R package “GSVA” was used to investigate differences in biological signaling and RNA modification patterns [39]. The gene set “c2.cp.kegg.v7.4.symbols” for GSVA analysis was obtained from the MSigDB database. Adjusted P-values ≤ 0.05 were known as significant. We used the R package “clusterProfiler” for performing functional annotation for RNA modification-related genes with |log2FC| > 0.1 and adjusted P-values ≤ 0.05 [40].

### Evaluation of TME cell infiltration

The ssGSEA (single-sample gene-set enrichment analysis) algorithm was used to quantify the relative abundance of each cell infiltrate in the GC TME. Gene sets for marking each TME infiltration cell type were obtained from Charoentong et al., which consisted of various human immune cell subtypes, including activated B cells, activated CD4 T cells, activated CD8 T cells, activated dendritic cells, macrophages, mast cells, natural killer T cells, and regulatory T cells (Supplementary Table 7) [41, 42]. Enrichment scores were calculated using ssGSEA analysis and represent the relative abundance of each TME infiltrating cell per sample (Supplementary Tables 8, 19).

### Association between RNA modification and cell signaling

Correlation analysis was performed to reveal the association between RNA modification patterns and cancer cell signaling. Gene sets of related biological pathways involved in this analysis were obtained from Mariathasan et al., including EMT markers EMT1/2/3, pan-fibroblast TGFb response signature (Pan-F-TBRS), Angiogenesis, antigen processing machinery (APM), CD8 T effectors and immune checkpoints [43]. Other gene sets used were from significantly activated signaling pathways in the GSVA analysis and DEGs between different WM_Cluster enriched pathways following KEGG analysis (Supplementary Tables 11, 12, 20).

### Immune and stromal scores

We used the ESTIMATE method to calculate stromal and immune scores and to analyze tumor purity for each patient using the R package “estimate”.

### Identification of DEGs

We subdivided RNA modification patterns according to the expression of 26 RNA modification “writers”. We also used the R package “limma”‘ the empirical Bayesian approach [44]. P-values ≤ 0.005 were deemed significant criteria for determining DEGs (Supplementary Table 13).

### KEGG enrichment analysis

We performed DEGs’ enrichment analysis and functional annotation by means of the R packages “clusterProfiler” and “org.hs.eg.db”. R packages “enrichplot” and “ggplot2” were used to construct bar plots following KEGG analysis.

### WM_Score scoring system construction

PCA was used for the construction of the WM_Score scoring model. Establishment of a matrix for the expression of DEGs was done in all patients. We selected PC1 and PC2 as signature scores. WM_Scores were calculated for each patient using a comparable method to GGI [45]:


WM_Score=∑(PC1i + PC2i)

where i denotes the expression of DEGs between different RNA modification patterns. A cutoff value was determined using the R package “survminer” according to WM_Scores and the survival status of the patients. We divided patients into high and low WM_Score groups in accordance with the cutoff value.

### Acquisition of stemness indexes

Profiles of the stemness index were downloaded from previous studies [46]. Four stemness indices were assessed through the analysis of transcriptomic and epigenetic feature sets.

### Weighted Gene Co-Expression Network construction and the identification of clinical trait-related modules

The R package “WGCNA” could conduct weighted gene co-expression network analysis for the 1801 DEGs according to different RNA modification patterns [47]. Patients were initially filtered in the TCGA-STAD cohort to remove outliers according to the expression of DEGs. We constructed a weighted network. We employed the “TOMSimilarity” function to transform the adjacency matrix into a TOM, which measures the network connectivity of genes. We next plotted the dynamic dendrogram using the “dynamicMods” function with minModuleSize = 60 to identify modules. Finally, we calculated GS and MM.

### Analysis of Copy number variant (CNV)

GISTIC 2.0 was employed to detect the common copy number alterations in all samples based on SNP6 CopyNumber segment data [48]. Masked Copy Number Segment data from the TCGA was first downloaded and disposed of using an R code. Disposed of data was uploaded onto the online analysis tool GenePattern (https://cloud.genepattern.org/gp/pages/login.jsf) for GISTIC analysis with confidence levels set at 0.95. Finally, the R package “maftools” was applied to visualize the GISTIC analysis.

### Relationship between WM_Score and miRNA regulation

Data regarding miRNA expression was downloaded from the TCGA-STAD cohort. DEmiRNAs between high and low WM_Score groups were determined using the R package “edgeR”. MiRNAs with |log2FC| >1 and adjusted P-values ≤ 0.05 were considered significantly differentially expressed. We screened DEGs with |log2FC| >1 and adjusted P-values ≤ 0.05 between WM_Score high and low groups using “edge R”. The miRTarBase (https://miRTarBase.cuhk.edu.cn/) database was used to predict target genes amongst the DEGs of DEmiRNAs [49]. KEGG enrichment analysis was performed. Finally, R packages “magrittr”, “tidyverse”, “crosslink” and “ggplot2” were employed to construct a circLink plot to directly reveal differences in DEmiRNA-targeted signaling pathways between high and low WM_Score GC patients.

### Association between WM_Score and APA events

APA data of STAD were obtained from the synapse database (https://www.synapse.org/#!Synapse:syn11511914). Changes in APA usage in each tumor were quantified as a change in the Percentage of Distal polyA site Usage Index (PDUI), which can identify 3’UTR lengthening (positive index) or shortening (negative index). The R package “edgeR” was used to distinguish transcripts with significantly differential PDUI values between high and low WM_Score patients.

### Association between WM_Score and A-to-I editing

The profile of A-to-I RNA editing frequency in STAD was obtained from the synapse database (https://www.synapse.org/#!Synapse:syn4382531). We used the R package “edgeR” to screen sites with significantly different A-to-I RNA editing frequencies between high and low WM_Score patients.

### Survival analysis for WM_Score-related APA events and A-to-I editing

Univariate Cox regression analysis was performed to identify survival-related PDUI of transcripts and A-to-I editing frequencies of gene sites. R package “survival” and “survminer” was applied to plot Kaplan-Meier curves. P ≤ 0.05 in the log-rank test was considered statistically significant.

### Data from immunotherapeutic cohorts

Gene expression profiles and clinical information of immunotherapeutic cohorts that could be publicly obtained were analyzed. Four immunotherapeutic cohorts were included: (1) pembrolizumab treatment for metastatic gastric cancer [50]; (2) IMvigor210 cohort, mUC treated with atezolizumab [43]; (3) melanoma patients treated with anti-PD1 ICB Nivolumab or Pembrolizumab [51]; (4) GSE78220 cohort, melanomas undergoing pembrolizumab therapy [52]. Basic information about these cohorts is listed in Supplementary Table 1.

### Validation of expression levels

Expression analysis was performed. Data were downloaded from reverse-phase protein arrays (RPPAs) of the 392 STAD patients in the TCGA. We performed correlation analysis between biological signaling pathways and the WM_Score [53]. Pathologic immunohistochemistry images of RNA modification “writers”, immune-activation-related genes, TGFβ-EMT pathway-related genes, and immune-checkpoint-related genes of GC patients were further performed using the Human Protein Atlas (HPA, https://www.proteinatlas.org/). Staining of each gene within the pathological tissue was obtained using the same antibodies [54].

### Statistical analysis

All statistical analyses were generated by R version 4.1.3. The R package “maftools” was utilized to plot waterfall maps of the mutation landscape of 26 “writers” in the whole TCGA-STAD cohort and genes for targeted therapy in high and low WM_Score groups. R package “pheatmap” was used to generate heat maps of the methylation profile of the “writers”. R package “RCircos” was used to present the copy number variation landscape of 26 RNA modification “writers” on 23 pairs of chromosomes. Spearman correlation analysis was applied to calculate the correlation coefficient of expression of these “writers” and correlation tests to evaluate the P-values. Wilcoxon tests were used to perform comparisons between the two groups, whilst Kruskal-Wallis tests were performed for the analysis of three or more groups. The Univariate Cox regression model was adopted to calculate hazard ratios (HR) for single RNA modification “writers” in GC and for WM_Scores across pan-cancer types. The R package “forestplot” was used for generating forest maps for visualization of the data. We used the Kaplan-Meier method. R package “survminer” was used to determine the cut-off point of subgroups for each dataset. The function “surv-cutpoint” was used to dichotomize the WM_Score and divide patients into high and low WM_Score groups. WM_Score’s specificity and sensitivity were assessed through ROC curves generated using the R package “survivalROC”.

## Supplementary Material

Supplementary Figures

Supplementary Table 1

Supplementary Table 2

Supplementary Table 3

Supplementary Table 4

Supplementary Table 5

Supplementary Table 6

Supplementary Table 7

Supplementary Table 8

Supplementary Table 9

Supplementary Table 10

Supplementary Table 11

Supplementary Table 12

Supplementary Table 13

Supplementary Table 14

Supplementary Table 15

Supplementary Table 16

Supplementary Table 17

Supplementary Table 18

Supplementary Table 19

Supplementary Table 20
