# Supplementary material for: Cross-talk of RNA modification "writers" describes tumor stemness and microenvironment and guides personalized immunotherapy for gastric cancer
Source: Aging (Albany NY). 2023 Jun 14;15(12):5445–81. doi: 10.18632/aging.204802 (PMC10333070; doi:10.18632/aging.204802)
Supplement: Supplementary Figures [file aging-15-204802-s001.pdf]

SUPPLEMENTARY FIGURES

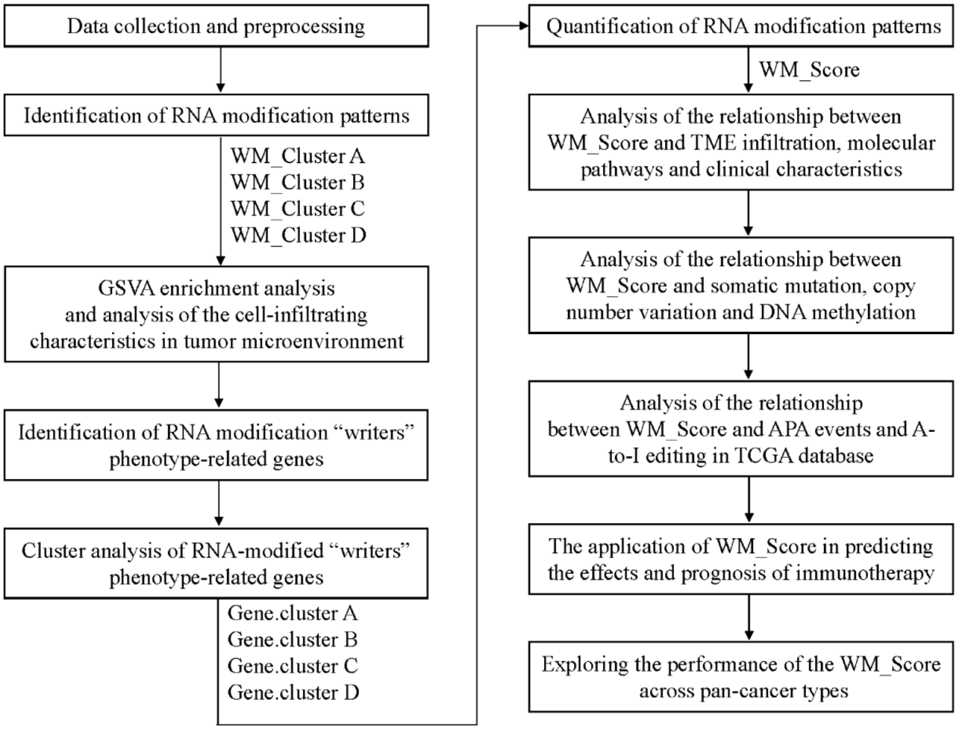

Supplementary Figure 1. Overview design of this study. This is the workflow of the steps performed in the study.

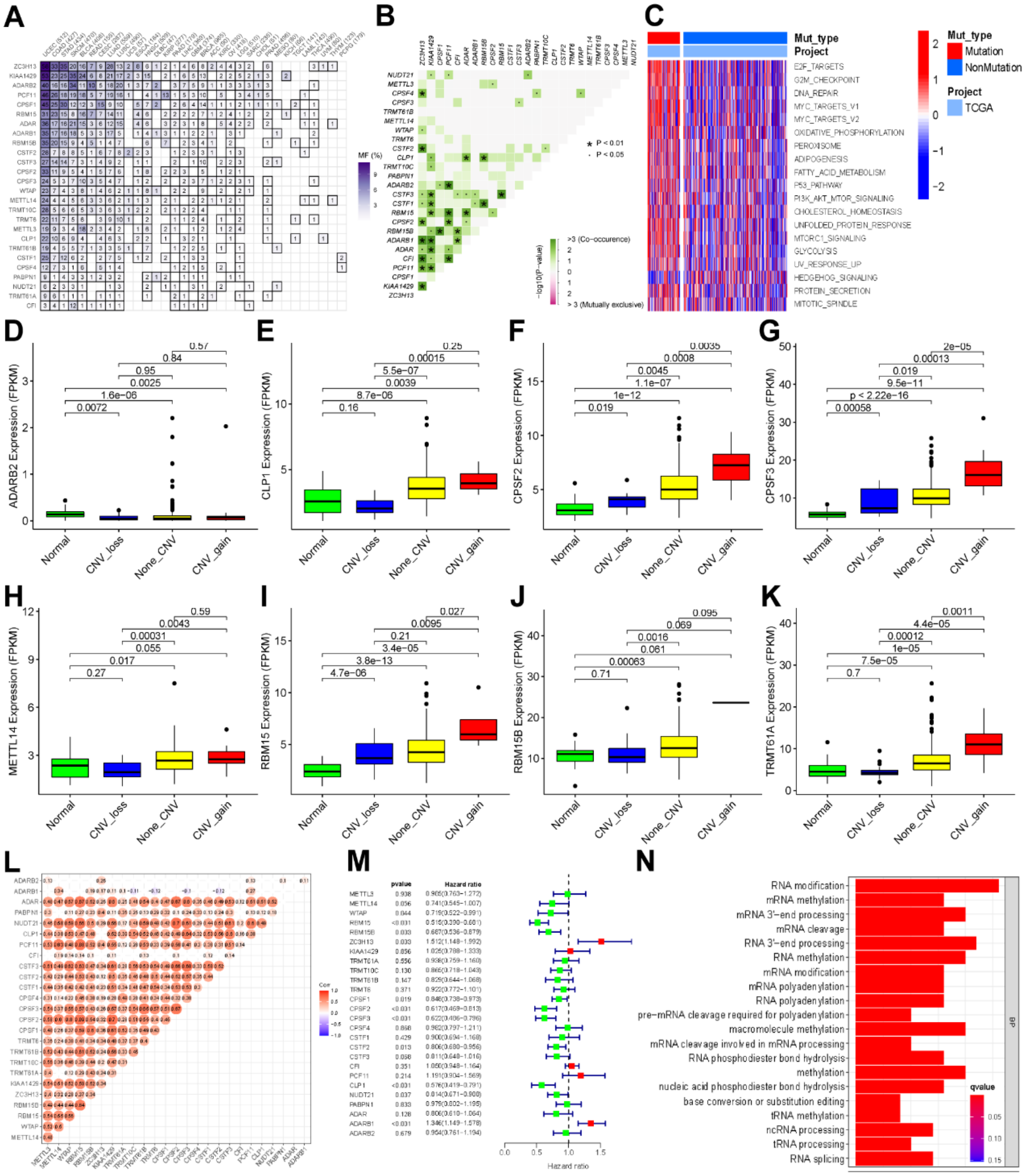

Supplementary Figure 2. Genetic alteration, co-expression and prognostic analysis of the 26 RNA modification "writers" in gastric cancer. (**A**) The mutation frequency of RNA modification "writers" among 33 cancer types in TCGA. The horizontal axis represents cancer types, and the numbers of samples are given in the parentheses. The vertical axis lists the names of the genes. (**B**) The mutation co-occurrence and exclusion analysis for the 26 RNA modification "writers". Co-occurrence, green; exclusion, purple. (**C**) GSVA enrichment analysis show the differentially activated biological pathways between patients with mutation of one or more of the 26 writers and without mutation. (**D**–**K**) The expression of *ADARB2* (**D**), *CLP1* (**E**), *CPSF2* (**F**), *CPSF3* (**G**), *METTL14* (**H**), *RBM15* (**I**), *RBM15B* (**J**) and *TRMT61A* (**K**) in different CNV status of patients. Kruskal-Wallis test was used for statistical analysis and p-values are marked above the boxes. (**L**) Spearman correlation analysis of the RNA modification "writers" expression. The correlation coefficients with $p < 0.05$ are marked in the circles, the sizes of which reflect the strength of correlation. (**M**) Forest plot shows the prognostic significance of the writers by a univariate Cox regression model. Hazard ratio < 1 represents protective factors for survival while hazard ratio > 1 represents risk factors for survival. (**N**) GO-BP enrichment analysis for the 26 RNA modification writers. The length of the bars represents the number of genes enriched.

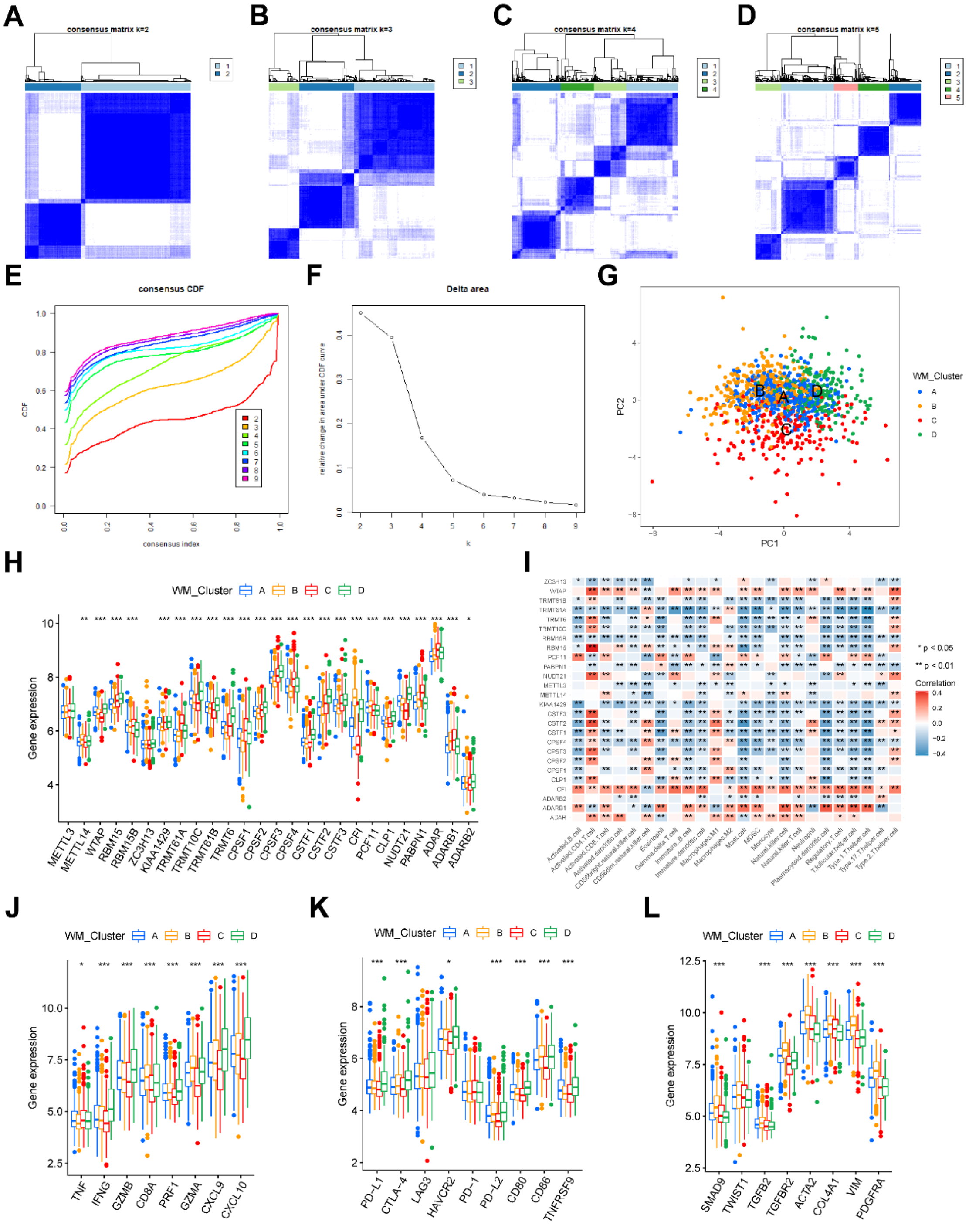

Supplementary Figure 3. Unsupervised clustering of the 26 RNA modification "writers" in the meta-GEO cohort. (**A**–**D**) Consensus matrices of the meta-GEO cohort for k = 2-5. (**E**) Consensus CDF curve for k = 2-9. (**F**) Relative change in area under CDF curve for k = 2-9. (**G**) Principal component analysis for the transcription of the 26 writers in patients with different RNA modification patterns. (**H**) The expression levels of 26 RNA modification writers in four types of RNA modification patterns. (**I**) The correlation between 24 TME infiltration cells and 26 RNA modification writers. Red means positive correlation while blue means negative correlation (Spearman's Correlation test). (**J**–**L**) The expression levels of some immune-activation related genes (**J**), immune-checkpoint related genes (**K**) and TGFβ- EMT pathway-related genes in the four types of RNA modification patterns. In both (**H**–**K**) the asterisks represent the statistical p-value (*P < 0.05; **P < 0.01; ***P < 0.001).

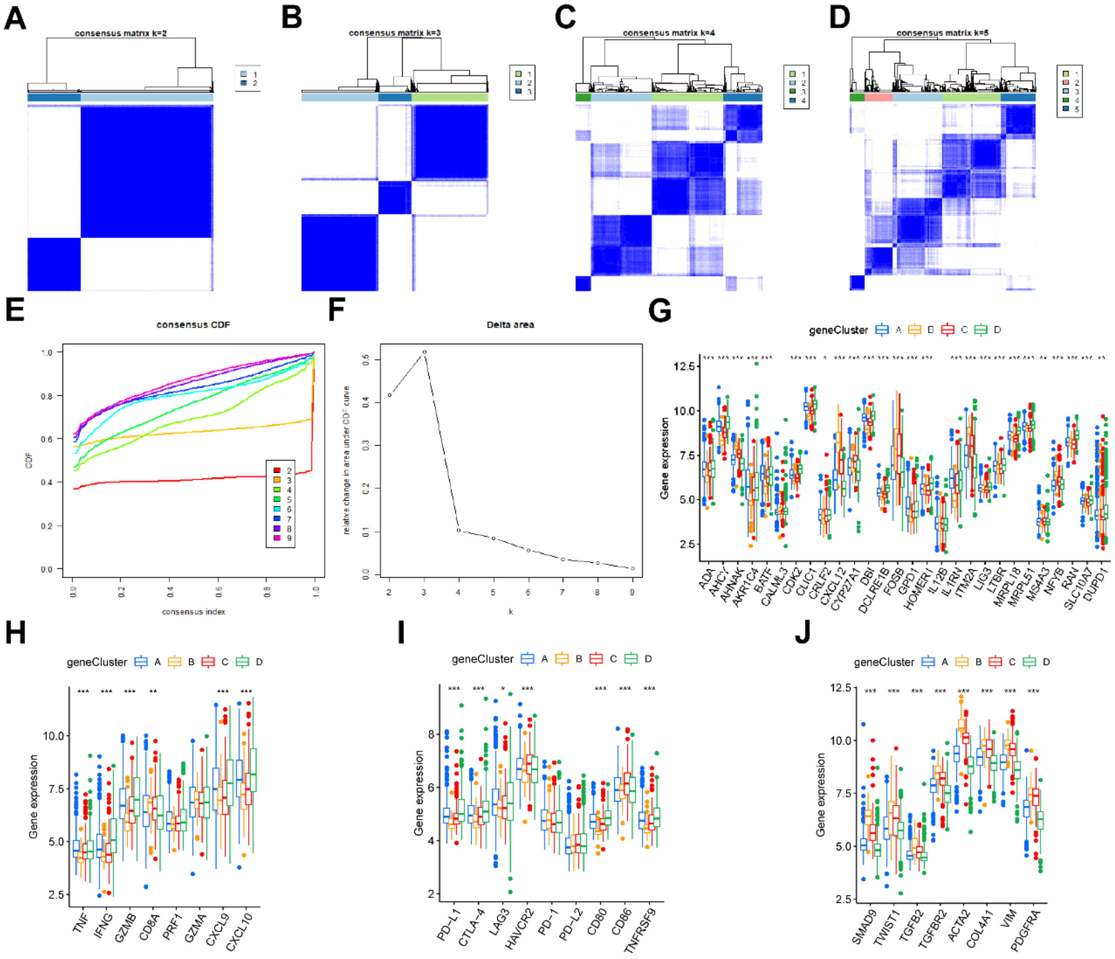

Supplementary Figure 4. The known immune or stromal-related gene signatures in the characterized distinct gene clusters. (**A**–**D**) Unsupervised clustering of 1801 RNA modification-related DEGs and consensus matrices of the meta-GEO cohort for k = 2-5. (**E**) Consensus CDF curve for k = 2-9. (**F**) Relative change in area under CDF curve for k = 2-9. (**G**–**J**) The expression levels of some T cell function enhancers (**G**), immune-activation related genes (**H**), immune-checkpoint related genes (**I**) and TGFβ- EMT pathway-related genes (**J**) in the four gene clusters (*P < 0.05; **P < 0.01; ***P < 0.001).

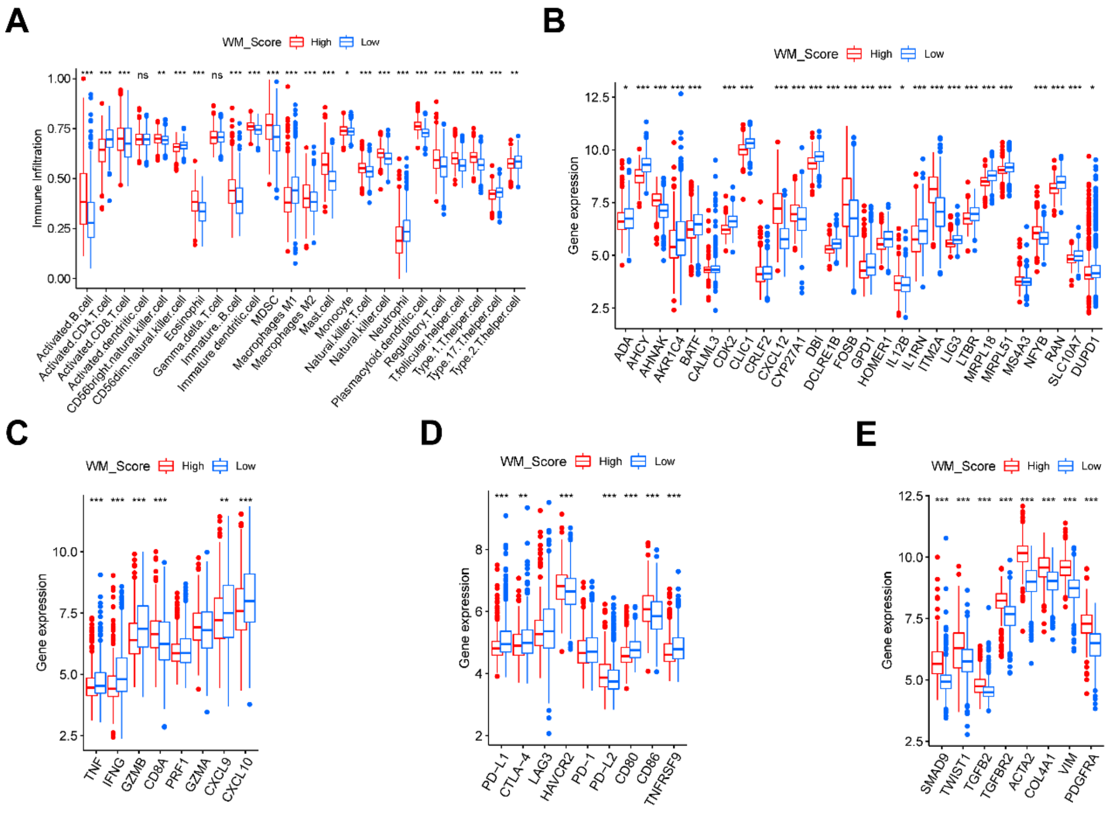

Supplementary Figure 5. The relationship between TME cell infiltration, known gene signatures and WM_Score. (**A**–**E**) The relative abundance of 24 TME infiltrating cells (**A**), expression levels of T cell function enhancers (**B**), immune-activation related genes (**C**), immune-checkpoint related genes (**D**) and TGFβ- EMT pathway-related genes (**E**) in WM_Score high and low groups of patients (*P < 0.05; **P < 0.01; ***P < 0.001).

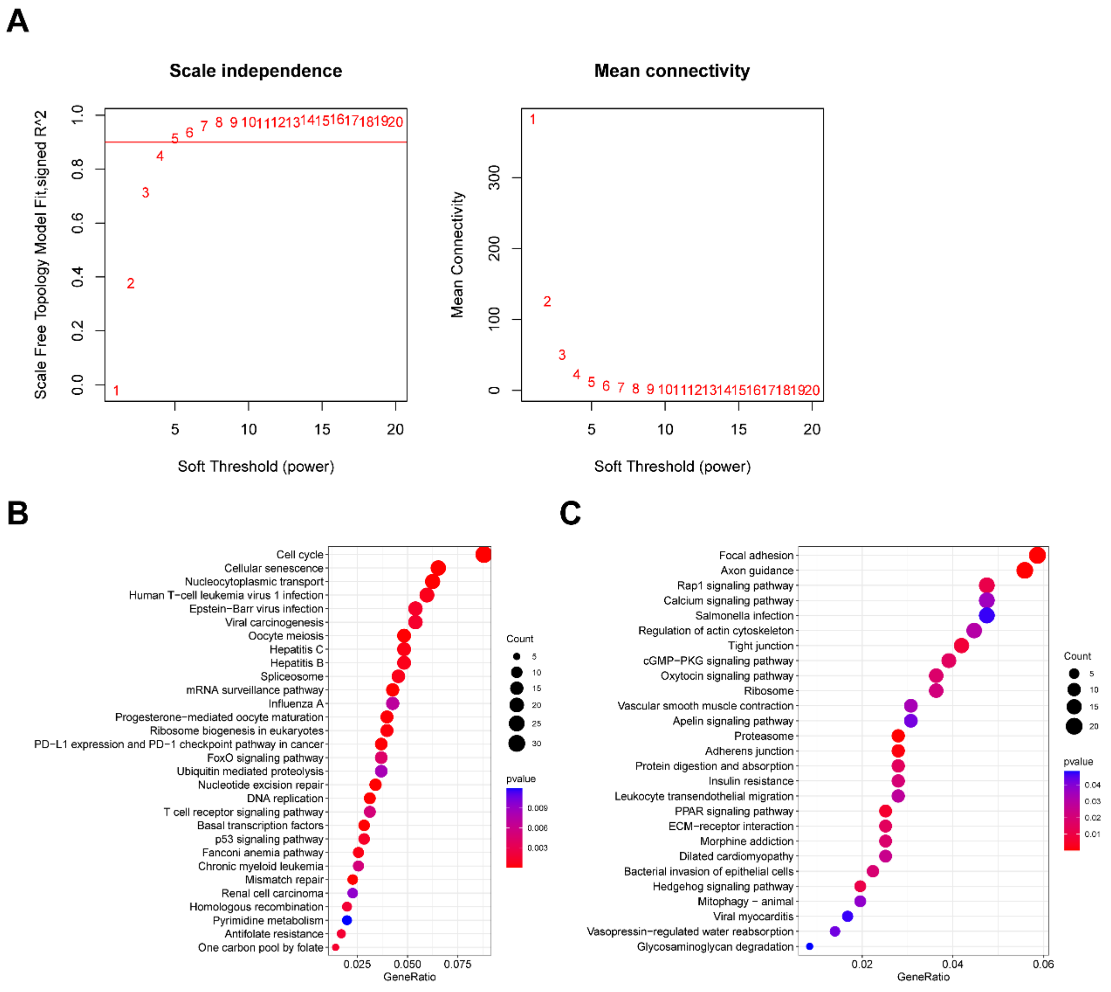

Supplementary Figure 6. The genes in different modules in WGCNA enriched in different pathways. (**A**) Detecting the optimal soft-thresholding power. When the power value is five, the degree of independence was > 0.9 for the first time. (**B**, **C**) KEGG enrichment analysis for the genes in modules of Figure 6H marked with turquoise (**B**) and blue (**C**). The x-axis indicates gene ratio within each KEGG term.

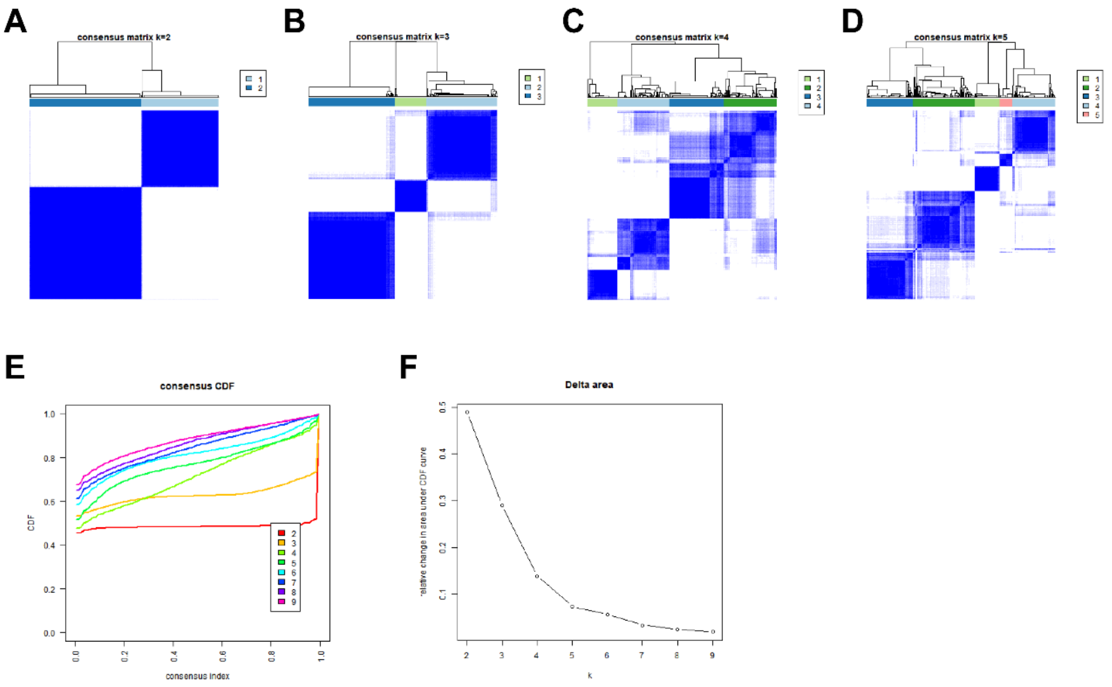

Supplementary Figure 7. Identification of DNA methylation subtypes of patients in TCGA-STAD cohort by unsupervised clustering. (**A**–**D**) Unsupervised clustering of the DNA methylation profile in TCGA-STAD cohort and consensus matrices for k = 2-5. (**E**) Consensus CDF curve for k = 2-9. (**F**) Relative change in area under CDF curve for k = 2-9.

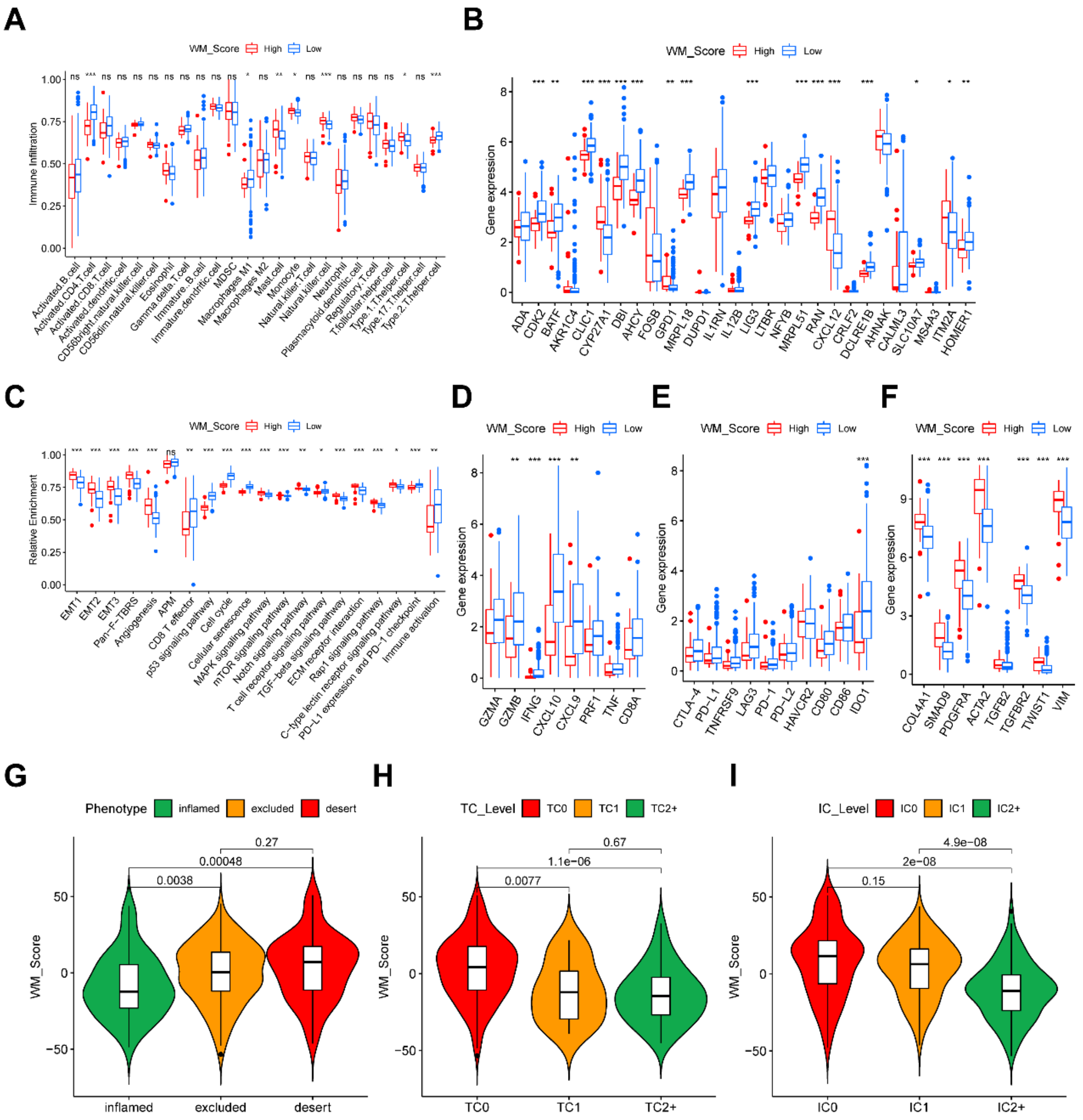

Supplementary Figure 8. The relationship between TME cell infiltration, signal pathways, known gene signatures and WM_Score in IMvigor210 cohort. (**A**–**F**) The relative abundance of 24 TME infiltrating cells (**A**), expression levels of T cell function enhancers (**B**), biological pathway gene signatures (**C**), immune-activation related genes (**D**), immune-checkpoint related genes (**E**) and TGFβ-EMT pathway-related genes (**F**) in WM_Score high and low groups of patients (*P < 0.05; **P < 0.01; ***P < 0.001). (**G**–**I**) Violin plots show the WM_Score in patients with different tumor immune phenotype (**G**), tumor cells (**H**) and immune cells (**I**) in IMvigor210 cohort.
