# Supplementary material for: Cross-talk of RNA modification "writers" describes tumor stemness and microenvironment and guides personalized immunotherapy for gastric cancer
Source: Aging (Albany NY). 2023 Jun 14;15(12):5445–81. doi: 10.18632/aging.204802 (PMC10333070; doi:10.18632/aging.204802)
Supplement: Supplementary Table 1 [file aging-15-204802-s002.pdf]

Supplementary Table 1. Basic information about the datasets included in this study.

Accession number	Publication	PMID	Platform	Number of patients	Stage	Gender	Survival data
GEO: GSE15459	Oncogenic pathway combinations predict clinical prognosis in gastric cancer.	19798449	GPL570	200	I/II:60 III/IV:132	Female:67 Male:125	OS
GEO: GSE34942	Identification of molecular subtypes of gastric cancer with different responses to PI3-kinase inhibitors and 5-fluorouracil.	23684942	GPL570	56	I/II:22 III/IV:31	Female:20 Male:36	OS
GEO: GSE57303	Whole genome gene copy number profiling of gastric cancer identifies PAK1 and KRAS gene amplification as therapy targets.	24935174	GPL570	70	I/II:12 III/IV:58	Female:18 Male:52	OS
GEO: GSE62254	Molecular analysis of gastric cancer identifies subtypes associated with distinct clinical outcomes.	25894828	GPL570	300	I/II:127 III/IV:173	Female:101 Male:199	OS
GEO: GSE84437	Deconvolution of diffuse gastric cancer and the suppression of CD34 on the BALB/c nude mice model.	32293340	GPL6947	433	I/II:49 III/IV:384	Female:137 Male:296	OS
GEO: GSE78220	Genomic and Transcriptomic Features of Response to Anti-PD-1 Therapy in Metastatic Melanoma.	26997480	GPL11154	27	NA	Female:8 Male:19	OS
Kim GC cohort	Comprehensive molecular characterization of clinical responses to PD-1 inhibition in metastatic gastric cancer	30013197	Illumina HiSeq 2000	45	NA	NA	NA
IMvigor210	TGF-β attenuates tumour response to PD-L1 blockade by contributing to exclusion of T cells	29443960	Illumina HiSeq 2500	348	I/II:213 III/IV:135	Female:76 Male:272	OS
David Liu cohort	Integrative molecular and clinical modeling of clinical outcomes to PD1 blockade in patients with metastatic melanoma	31792460	Illumina HiSeq 2501	144	NA	Female:60 Male:84	OS

TCGA	Normal samples	Tumor samples	Stage	Gender	Mutation	CNV	APA number	A-I number	Survival data
TCGA-STAD	32	443	I/II:161 III/IV:187 unknown:2 3	Female:133 Male:238	433	440	318	318	371
TCGA-Pan-cancer	NA	9583	NA	NA	NA	NA	NA	NA	9583
